# Chemically‐Disordered Transparent Conductive Perovskites With High Crystalline Fidelity

**DOI:** 10.1002/advs.202509868

**Published:** 2025-07-12

**Authors:** Saeed S. I. Almishal, Pat Kezer, Jacob T. Sivak, Yasuyuki Iwabuchi, Sai Venkata Gayathri Ayyagari, Saugata Sarker, Matthew Furst, Gerald Bejger, Billy Yang, Simon Gelin, Nasim Alem, Ismaila Dabo, Christina M. Rost, Susan B. Sinnott, Vincent Crespi, Venkatraman Gopalan, Roman Engel‐Herbert, John T. Heron, Jon‐Paul Maria

**Affiliations:** ^1^ Department of Materials Science and Engineering The Pennsylvania State University University Park PA 16802 USA; ^2^ Department of Electrical Engineering University of Michigan Ann Arbor MI 48109 USA; ^3^ Department of Chemistry The Pennsylvania State University University Park PA 16802 USA; ^4^ Department of Physics The Pennsylvania State University University Park PA 16802 USA; ^5^ Department of Materials Science and Engineering Virginia Polytechnic Institute and State University Blacksburg VA 24061 USA; ^6^ Institute for Computational and Data Sciences The Pennsylvania State University University Park PA 16802 USA; ^7^ Paul‐Drude Institute for Solid State Electronics Hausvogteiplatz 5–7 10117 Berlin Germany; ^8^ Department of Materials Science and Engineering University of Michigan Ann Arbor MI 48109 USA

**Keywords:** cluster expansion, DFT, disorder, electron correlation, high‐entropy oxides, perovskites, TEM, thin films, transparent conductors, XPS, XRD

## Abstract

This manuscript presents a working model linking chemical disorder and transport properties in correlated‐electron perovskites with high‐entropy formulations and a framework to actively design them. This work demonstrates this new learning in epitaxial Sr_x_(Ti,Cr,Nb,Mo,W)O_3_ thin films that exhibit exceptional crystalline fidelity despite a diverse chemical formulation where most *B*‐site species are highly misfit with respect to valence and radius. X‐ray diffraction, X‐ray photoelectron spectroscopy, and transmission electron microscopy confirm a unique combination of chemical disorder and structural perfection in thin and thick epitaxial layers. This combination produces an optical transparency window that surpasses that of the constituent end‐members in the UV and IR, while maintaining relatively low electrical resistivity. This work addresses the computational challenges of modeling such systems and investigate short‐range ordering using cluster expansion. These results showcase that unusual *d*‐metal combinations access an expanded property design space that is predictable using end‐member characteristics and their interactions – though unavailable to them – thus offering performance advances in optical, high‐frequency, spintronic, and quantum devices.

## Graphical abstract

Kinetically arrested, chemically disordered perovskite thin films exhibit exquisite crystalline fidelity, broad IR–UV transparency, and low resistivity, despite incorporating five mismatched 3d–5d cations, including Cr and W. This study unites electronic correlation, cation diversity, valence complexity, and disorder—enabling new transparent conductors and paving the way toward novel quantum and spintronic applications.

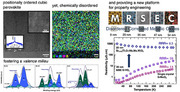

## 1 Introduction

Correlated materials host exotic phenomena induced by electron‐electron interactions such as high‐temperature superconductivity and quantum magnetism, possibly enriched by topological phenomena. These emergent properties could revolutionize future technologies such as quantum computation.^[^ ^1^, ^7^ ^]^ Correlated oxides in the A*B*O_3_ perovskite structure attract significant attention because their structure and properties are tunable through cation substitution, and they can be integrated into functional heterostructures and superlattices.^[^ ^1^, ^8^ ^]^ Sr_x_ *B*O_3_ cubic perovskites are particularly interesting for high electrical conductivity especially when early transition metal cations with n*d* ^1^ and n*d* ^2^ electronic configurations occupy the *B*‐site.^[^ ^1^, ^2^, ^9^, ^14^ ^]^ Remarkably, the Sr_x_ *B*O_3_ cubic perovskites remain paramagnetic metals (with no metal‐to‐insulator transitions near room temperature) with an optical transparency window that results from electron correlation. Sr_x_VO_3_, Sr_x_NbO_3_ and Sr_x_MoO_3_ are thus extensively studied as transparent conductors.^[^ ^2^, ^9^, ^11^, ^13^ ^]^ In contrast, **Sr_x_CrO_3_** (with 3d^3^ Cr^3^⁺ on the B‐site) and **Sr_x_WO_3_** (with heavy 5d W cations) remain largely unexplored due to their metastability in the cubic perovskite phase. Yet, as we detail in Section 2, **Sr_x_CrO_3_** holds promise for enhancing correlation and electrical conductivity, while **Sr_x_WO_3_** offers a platform for probing strong spin‐orbit coupling (SOC)—a key driver of emerging quantum phenomena such as magnetocrystalline anisotropy, non‐collinear magnetism, the anomalous and spin Hall effects, spin Seebeck effect, and Rashba–Edelstein effect^[^ ^7^, ^15^, ^18^ ^]^ Despite their promise, all Sr_x_ *B*O_3_ family members thermodynamically prefer insulating phases like scheelite and barite unless heroically engineered with significant vacancies on the *A*‐site and grown under highly reducing conditions.^[^ ^10^, ^19^, ^20^ ^]^

High‐entropy oxides (HEOs) can stabilize structure‐formulation combinations with unusual coordination and valence,^[^ ^21^, ^23^ ^]^ thus a possible new route to prepare early‐transition‐metal Sr_x_ *B*O_3_ perovskites. HEOs maximize chemical configurational entropy on equivalent cation sublattice sites while maintaining positional order, which tends to thermodynamically favor homogeneous high‐symmetry phases. Such entropy‐favored phases can be further promoted by quenching energetic plasma adatoms on a comparatively cool substrate to capture a high effective‐temperature structure and kinetically arrest further transitions to equilibrium.^[^ ^24^, ^26^ ^]^ As such, combining many‐cation chemical formulations with far‐from‐equilibrium synthesis methods boosts both entropy and temperature, potentially realizing crystals that violate Muller and Roy's structure field maps while retaining high crystalline fidelity and significant chemical disorder (Taxonomy: Note S1, Supporting Information).^[^ ^21^, ^22^, ^25^, ^27^, ^30^ ^]^ We therefore hypothesize that a Sr_x_ *B*O_3_ formulation with many cations on the *B*‐site can promote the high‐symmetry cubic perovskite phase, solvating a diverse palette of early and heavy transition metals and unlocking novel correlation‐disorder induced properties. This approach may enable transparent conductors with a more tunable ultraviolet‐to‐infrared (UV‐to‐IR) optical response, heightened SOC with controllable resistivity, spin‐orbit torque devices, and high‐energy plasmonic systems.^[^ ^7^, ^11^, ^18^, ^31^ ^]^ To initiate learning in disordered correlated perovskite metals, we explore the relationships linking optical transparency and electrical conductivity. We first formulate design rules that identify Sr_x_(Ti,Cr,Nb,Mo,W)O_3_ as a candidate composition in this space, then use high kinetic energy synthesis to realize high‐quality Sr_x_(Ti,Cr,Nb,Mo,W)O_3_ films that are practically metastable, though far from equilibrium under room conditions. In parallel, we develop a cluster expansion model to predict tendencies for short‐range order in such materials as a function of temperature. Through this process we demonstrate an electrical conductivity‐optical transparency combination that rivals endmember possibilities.

## 2 Designing Chemical Disorder on the Perovskite B‐Site for Optical Transparency and Electrical Conductivity

**Figure 1:**
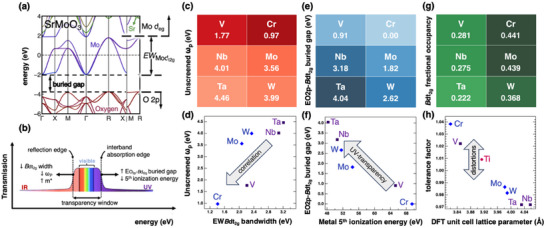
B‐cation design criteria. a) SrMoO_3_ calculated band structure, b) engineering the transparency window, c) unscreened plasma frequency map for early transition metals, d) the relation between bandwidth W*_B_*_dt2g_ and plasma frequency ω_p_: greater d‐orbital overlap leads to lower electron‐electron correlation, a larger EW*B*_t2g_ and a higher energy plasma frequency (we rely on EW*_B_*_dt2g_ and ω_p_ as correlation indicators rather than the typical renormalization factor Z_k_) e) map for buried energy gap *E_O2p‐Bt2g_* defined as the gap between the highest energy lying O_2p_ state and the lowest energy *B*dt_2g_ state, f) the inverse relation between the buried gap and the fifth ionization energy, g) dt_2g_ fractional occupancy map and h) tolerance factor vs the unit cell lattice parameters obtained from DFT relaxation. c–h) are calculated from supercells with anti‐ferromagnetic type‐G (AFM‐G) ordering, which better approximates strong correlation than do nonmagnetic (NM) unit cells (Note S2, Supporting Information).^[^ ^26^, ^27^ ^]^

We begin with a computational exploration of Sr*B*O_3_ endmember band structures using density functional theory (DFT) to inform predictions of electronic structure and properties in many‐component solid solutions. As an example, **Figure** **1**a presents the SrMoO_3_ band structure. Three dt_2g_ bands originate from the 4*d* orbital manifold with a EW_Modt2g_ bandwidth (where EW*_B_*_dt2g_ denotes dt_2g_ energy bandwidth for cation B, thus EW_Modt2g_ denotes dt_2g_ energy bandwidth for Mo). These bands are partially filled by Mo *d*‐electrons and energetically isolated from the O_2p_ bands, forming a *buried energy gap*, E_O2p‐Modt2g_ (where Eo_O__2p‐*B*dt2g_ denotes the burried energy gap for cation *B*, thus E_O2p‐Modt2g_ denotes the burried energy gap for the case of Mo on the B‐site). Figure 1b shows a transmission (%) versus energy schematic for a hypothetical conducting perovskite oxide. The transparency window is bounded by a reflection edge on the low‐energy side and an absorption edge on the high‐energy side. As we argue below, these physical properties are tied directly to the descriptor energies EW*_B_*_dt2g_ and E_O2p‐_ *_B_*_t2g_, respectively. The overall band structure of our perovskite endmembers share a similar form and exhibit descriptor energies that vary systematically with elemental periodic properties (Note S2, Supporting Information). In most general terms, a smaller EW*_B_*_dt2g_ implies smaller *d*‐orbital overlap, stronger correlation and thus a larger electron effective mass. This effectively redshifts the plasma frequency ω_p_ and the associated IR reflection edge; Figure 1c,d shows this periodic trend. Based on this criterion, SrVO₃ and SrCrO₃ have the strongest correlation and are predicted to enhance transparency in the low‐energy visible and IR regions.

On the opposite end, the *E*_O2p‐_ *_B_*_dt2g_ energy gap can be related to the high energy absorption edge, which originates from interband transitions between occupied O_2p_ bands and unoccupied *B*dt_2g_ states above the Fermi level (E_F_), where a larger *E*_O2p‐_ *_B_*_dt2g_ gap blue‐shifts the transparency window into the UV spectrum. We note that prior authors suggested that this edge scales with orbital size or electronegativity difference between *B* and O.^[^ ^9^, ^10^, ^11^ ^]^ This trend notably fails for W and Mo, for which electronegativity models predict UV transparency but experiment finds absorption. Our *E*_O2p‐_ *_B_*_dt2g_ descriptor, however, correctly assigns SrNbO_3_ as more transparent than SrMoO_3_ and SrWO_3_, and importantly, offers some chemical intuition given its relationship to the *B*‐site metal cation fifth ionization energy (E_i_^5^) which is shown in Figure 1f. For the present series, E_i_^5^ values follow the hierarchy: Cr > V > Mo > W > Nb > Ta. Decreasing E_i_^5^ values indicate a willingness for 5^+^ oxidation states, which in turn depopulates the dt_2g_ bands, opens the O_2p_‐*B*_dt2g_ band energy separation and blue‐shifts the high energy absorption edge. A correlation reduction, however, usually accompanies this blue shift which slightly reduces the near‐IR transparency. With these criteria the broadest transparency window with high conductivity is expected for SrNbO_3_.

*B*_dt2g_ filling is the critical design parameter to optimize carrier density, the fractional filling associated with each *B*‐site cation is listed in Figure 1g. In addition to carrier density, band filling fraction can also increase correlation effects (captured when enforcing antiferromagnetic type‐G ordering and is not observed in the nonmagnetic case, see Note S2, Supporting Information) and contribute to structural distortions from cubic perovskite by filling antibonding states as shown in Figure 1h. SrMoO_3_ with 44% Mo4d_t2g_ predicted occupancy, has the lowest experimentally‐reported electrical resistivity with the highest carrier concentration in this family.^[^ ^13^, ^16^, ^26^ ^]^ Following these trends based on individual *B*‐site cation end members, we hypothesize that cubic perovskites with high Cr and W concentrations will exhibit high electrical conductivity because *B*dt_2g_ filling could rival SrMoO_3_. Additionally, Cr and W, as crystallochemical misfits, could lift centrosymmetry at short length scales intensifying electronic correlations and spin‐orbit interactions.

With these theory trends in mind, we propose Sr_x_(Ti,Cr,Nb,Mo,W)O_3_ as the ideal host optimizing high conductivity, high transparency and high spin‐orbit coupling despite the significant configurational disorder. Ta and V are excluded due to tendencies for insulating behavior and toxicity respectively, while Ti is added to stabilize the perovskite structure. As shown in Figure 1h, Ti introduces the least structural distortions—based on the tolerance factor versus DFT‐calculated unit cell volume—making it the ideal B‐site cation for the cubic perovskite structure when Sr occupies the A‐site. We note that Cr, Nb, Mo, and W are not perovskite *B*‐site cations at ambient equilibrium, thus part two of our guiding hypothesis is entropy‐assisted, kinetically‐enabled stabilization of the essential perovskite structure under reduced conditions, for reasons articulated earlier.

## 3 Stabilizing The Chemically‐Disordered Cubic Perovskite Phase through Far‐From‐Equilibrium Thin Film Growth

We anticipate that establishing perovskite Sr(Ti,Cr,Nb,Mo,W)O_3_ requires quenching high kinetic energy adatoms and substrate epitaxial constraints. In addition, engineering *A*‐site vacancies to balance *B*‐cations with > 4^+^ net valence is also necessary. We therefore first prepare bulk ceramics with 0% to 25% Sr deficiency (Note S3, Supporting Information). These ceramics, sintered at 1400 °C, exhibit both scheelite and perovskite phases, but pulsed laser deposition at 850 °C in 50 mTorr Ar produces epitaxial perovskite material with high crystalline fidelity on multiple substrates. Processing details needed to stabilize perovskite films are discussed in Methods and Notes S4–S7, S13, Supporting Information. Ultimately, ceramic targets with 5% A‐site vacancies produce superior crystals and properties (Note S4, Supporting Information). We further investigate this composition, henceforth referred to as Sr_0.95_BO_3_.

**Figure 2:**
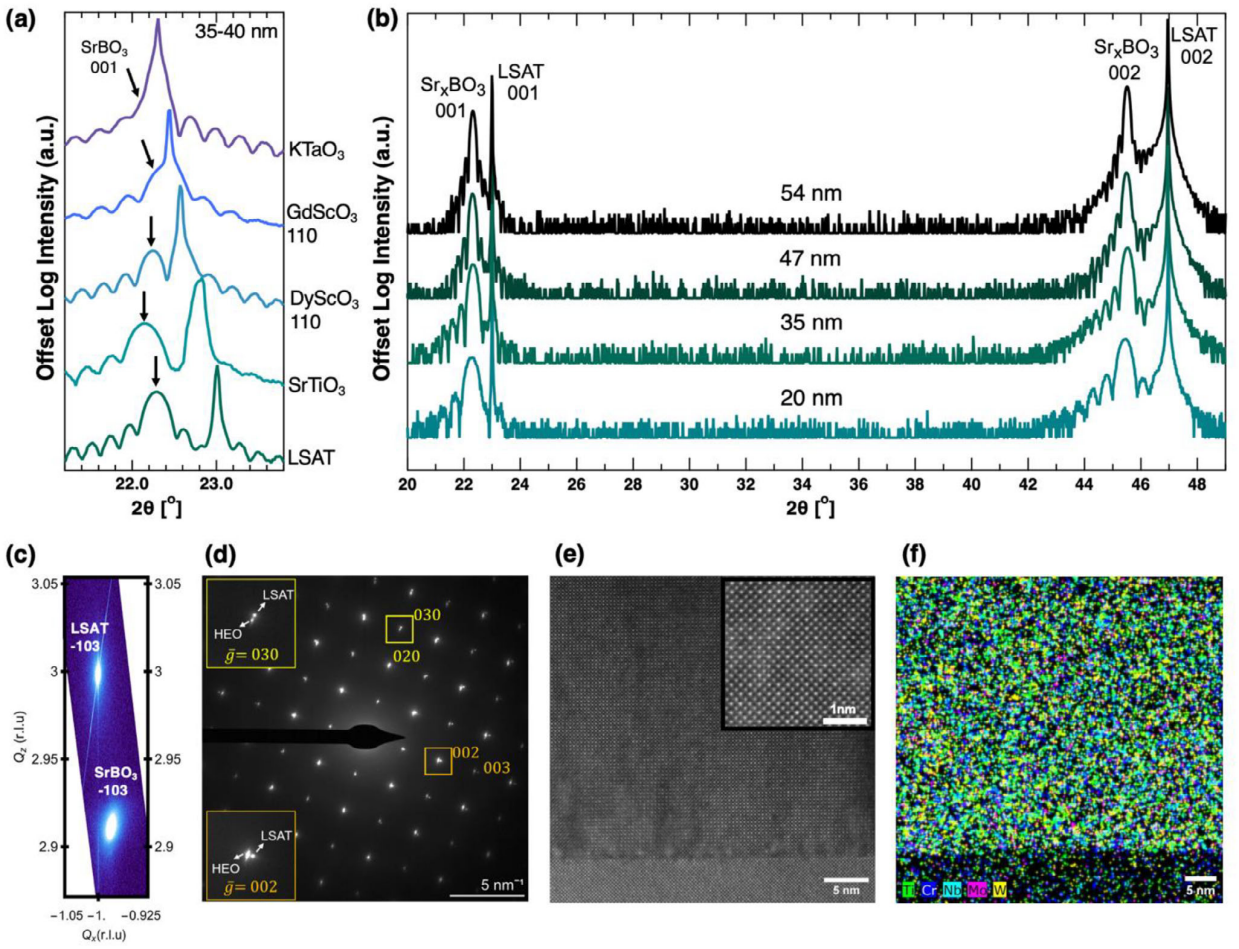
Sr_0.95_(Ti,Cr,Nb,Mo,W)O_3_ structural and chemical characterization. a) X‐ray diffraction patterns of the 001 peaks of films grown on different substrates in the 35‐40 nm thickness range, b) Wide high resolution X‐ray diffraction patterns of films grown on LSAT substrate with different thicknesses from 20 nm to 54 nm showing both the 001 and 002 peaks, c) the 35 nm film on LSAT reciprocal space map of the (‐103) reflections, d) 35 nm film on LSAT SAED pattern with insets highlighting the film and substrate diffraction spots (acquired from the [100] zone axis), e) drift‐corrected HAADF image of the same sample showing uniform cubic perovskite structure with a zoomed in inset, see Figure S13, Supporting Information for annular bright field STEM image, and f) representative EDX map showing the random distribution of B‐site cations. The EDX maps for the individual elements and their corresponding STEM image are included in Figure S14, Supporting Information. Note that (110) DyScO_3_ and (110) GdScO_3_ are equivalent to (001) pseudocubic orientation.

**Figure** **2**a depicts X‐ray diffraction (XRD) scans for Sr_0.95_BO_3_ films grown on different technologically relevant substrates: (001) (LaAlO_3_)_0.3_(Sr_2_TaAlO_6_)_0.7_ (LSAT), (001) SrTiO_3_, (110) DyScO_3_, (110) GdScO_3_ and (001) KTaO_3_ (Note S5, Supporting Information). The corresponding wide‐angle 2θ−ω XRD scans in Figure S8, Supporting Information show that only perovskite phase peaks are present. The strong diffraction peaks with pronounced Pendellösung fringes in Figure 2a indicate abrupt interfaces and smooth film surfaces, also confirmed by atomic force microscopy in Figure S9, Supporting Information. We choose LSAT substrates for thickness and electrical studies due to their insulating properties and ability to produce high‐quality crystals. Figure 2b illustrates four Sr_0.95_BO_3_ films grown on LSAT with increasing thickness up to 54 nm. All films exhibit high crystalline quality, as evidenced by the 001 and 002 peaks in Figure 2b and low mosaicity – rocking curve widths all below 0.05° using BBHD optics (see Note S6 and Figure S11, Supporting Information) – despite substantial chemical disorder and the fact that 4 *B*‐site cations prefer different equilibrium structures. This crystalline fidelity and epitaxial growth persists to 300 nm thickness with only partial broadening between 200 and 300 nm (Figure S12, Supporting Information). To the best of our knowledge, this is significantly thicker than previously reported end‐member thin films grown without a buffer layer (e.g., SrTiO_3_ buffer layer^[^ ^32^ ^]^), which typically show full relaxation or noticeable degradation in crystalline quality or compositional homogeneity beyond 60 nm.^[^ ^2^, ^9^, ^10^ ^]^ The asymmetric 1̅03 diffraction peak reciprocal space map (RSM) for the 35 nm film is shown in Figure 2c. From the RSM and out‐of‐plane symmetric scans, we calculate out‐of‐plane and in‐plane lattice constants of 3.983 Å (± 005Å) and 3.963 Å (± 005Å), respectively, yielding a c/a ratio ≈1.00. We calculate the film's intrinsic lattice parameter to be 3.967 Å, corresponding to a 2.5% lattice mismatch with the 3.87 Å LSAT substrate. Moreover, the RSM in Figure 2c suggests film relaxation, though it remains uncertain if this process is partial or complete.

Figure 2d shows selected area diffraction (SAED) for the 35 nm film, this pattern is most consistent with perovskite structure with a longer, on average, out‐of‐plane lattice parameter than the substrate (Figure S13, Supporting Information). Figure 2e shows a cross‐sectional high‐angle annular dark‐field (HAADF) image along the ⟨100⟩ zone. Intensity fluctuations indicate more disorder near the film substrate interface but they do not indicate a regular pattern of misfit dislocations despite the 2.5% lattice mismatch. Figure 2f shows a STEM‐EDX map highlighting *B*‐site cations with no indications of chemical segregation on the few‐nm length scale. Individual maps for each cation are shown in Figure S14, Supporting Information that further support this interpretation. Power‐dependent second harmonic generation (SHG) measurements were collected as a final probe of structure and symmetry. The analysis (shown in Note S7, Supporting Information) does not show SHG activity thus indicating a long range centrosymmetry.

**Figure 3:**
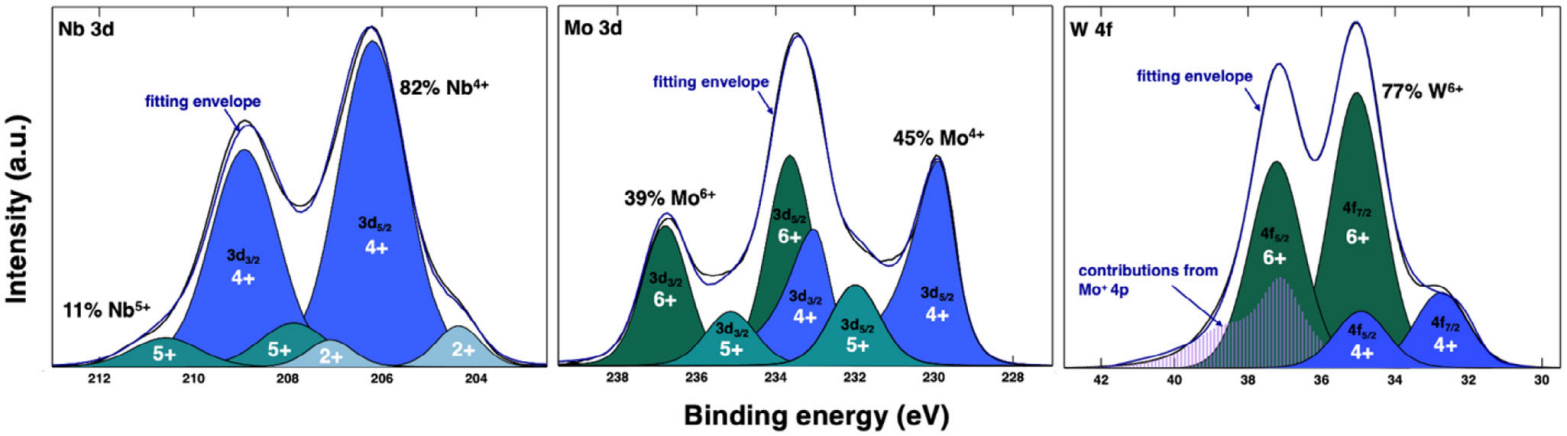
Sr_0.95_(Ti,Cr,Nb,Mo,W)O_3_ XPS fittings for Nb 3d (left), Mo 3*d* (middle) and W 4f (right) illustrating that the films exhibit magnified charge disproportionation. Notably, we cannot properly fit the W4*f* XPS spectra without accounting for Mo 4p peaks. This highlights the matrix effects present in such a chemically disordered system. Table S1, Supporting Information summarizes the oxidation states for all B‐cations with the A‐site being predominantly occupied with Sr^2+^ (Notes S8, S10, S11, Supporting Information).

Many *B*‐site cations present favor multiple valence states, especially when processed at high temperatures and oxygen‐lean conditions. As such, the chemical disorder is likely accompanied by a highly charge disordered environment where neighboring cations adopt oxidation states other than 4^+^ (For example, see Note S2 Figure S5, Supporting Information for Bader charges in binary B‐site compositions). A comprehensive set of X‐ray photoelectron spectroscopy (XPS) scans are conducted to resolve and quantify this likely valence milieu. The most informative scans and their fitting results are summarized in **Figure** **3**, with additional supporting information available in Note S8, Supporting Information. The most important findings include: (i) Nb is ≈82% Nb^4+^, 11% Nb^5+^, and 7% Nb^2^⁺ possibly suggesting *A*‐site occupancy. It is important to note, however, that Nb^3+^ cannot be excluded, as detailed in the Note S10, Supporting Information; (ii) Mo is 45% 4^+^, 39% 6^+^ and 16% 5^+^; (iii) W is 77% 6^+^ and 23% 4^+^; and (iv) Cr and Ti are predominanely 3^+^ and 4^+^, respectively. It is critical to acknowledge the valence quantification challenge in these complex mixtures. As shown in the Notes S8–S10, Supporting Information, reference sample measurements and extensive fitting procedures were used to meet this challenge. For corroboration, X‐ray absorption near edge structure (XANES) measurements were collected on a 200 nm film as detailed in Note S11, Supporting Information. XANES provides an effective average charge state between 5^+^ and 6^+^ for W, which is accessible due to its high absorption, consistent with our XPS results. The entire valence determination experiment can be tested for self‐consistency by applying charge neutrality. The details of this calculation are shown in Note S8 Table S2, Supporting Information, but the net result, when considering all measured valence values and the deliberate Sr vacancy concentration, is an equal and opposite positive and negative charge summation. Last, to connect the XPS‐derived valence states back to our design strategy in Section 1: although we have selected Cr, Mo, and W as nominal 4+ cations to fill the a*B*d_2tg_ ​​ bands, XPS and Bader analysis (Figure S5, Supporting Information) reveal that Cr reduces to 3+, while Mo and W undergo further oxidation. As a result, Cr likely contributes three conduction electrons, and Mo and W fewer than two; nevertheless, all three together exceed the possible electron contributions of V, Nb, and Ta, remaining consistent with our conductivity enhancement strategy based on *B*d_2tg_ ​filling.

## 4 Electron Transport Properties under Chemical Disorder

As shown above, multiple cations must access multiple valence states in these Sr_0.95_BO_3_ crystals to maintain charge neutrality. This creates an “electron scattering wilderness” superimposed on a lattice with remarkable crystalline fidelity, a combination that can promote flat temperature and frequency dependences to charge transport and optical responses, respectively.

### 4.1 Optical Properties

**Figure 4:**
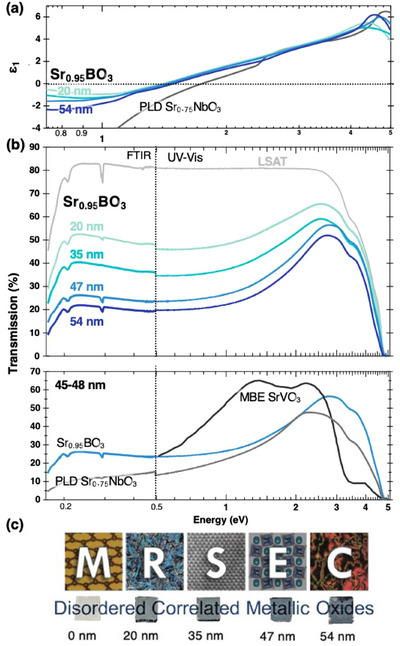
Sr_0.95_BO_3_ optical properties. a) shows the real part of the complex dielectric function of Sr_0.95_BO_3_ in comparison to our 48 nm PLD grown Sr_x_NbO_3_; b) upper part shows the corresponding optical transmission (%) from UV–vis and FTIR, while the lower panel shows the transmission of 47 nm Sr_0.95_BO_3_ film in comparison to our Sr_0.75_NbO_3_ and Zhang et al. MBE SrVO_3_ with comparable thicknesses; and c) depicts the real samples photograph of Sr_0.95_BO_3_ film series.

In **Figure** **4**, we show the optical properties of Sr_0.95_BO_3_ films grown on LSAT and compare them with our PLD‐grown Sr_x_NbO₃ (Note S9, Supporting Information), which is reported to have the widest transparency window among end‐members.^[^ ^9^, ^11^, ^12^ ^]^ Figure 4a shows the real part of the dielectric function (ε_1_) while Figure S21b,c, Supporting Information show the corresponding imaginary part of the dielectric function (ε_2_) and the extinction coefficient (k), respectively, all calculated from ellipsometry. The Sr_0.95_BO_3_ films ε_1_ and ε_2_ values are consistent across all thicknesses, with modest variations likely arising from stoichiometry, strain relaxation, surface scattering, and multiple internal reflections. ε_1_ crosses zero at ≈1.33 eV, this energy corresponds to a screened plasma frequency where ionic screening and interband electronic transitions introduce non‐Drude contributions to the energy dependence. This is comparable to the situation for SrVO₃, and red‐shifted compared to Sr_x_NbO₃ and SrMoO_3_^[^ ^2^, ^9^, ^12^ ^]^ This relatively low screened plasma frequency suggests strong electron correlation effects, likely arising from cations such as Cr and Mo, in combination with significant configurational disorder. In the context of perovskite conductors, ε_2_ and k of Sr_0.95_ *B*O_3_ are quite low in the IR, visible and UV regimes, with a sharp increase at higher photon energies attributed to *E*_O2p‐_ *_B_*_dt2g_ interband transitions. Figure S21d, Supporting Information shows k obtained from ultraviolet‐visible (UV–vis) spectroscopy, closely matching ellipsometry results and reinforcing our confidence in the measurements and fittings.

Figure 4b shows transmission spectra for SrBO_3_ as a function of film thickness from UV–vis and Fourier‐transform infrared spectroscopy (FTIR). Sr_0.95_BO_3_ films exhibit remarkable transparency in the IR and UV compared to the seminal molecular beam epitaxy (MBE) SrVO₃^[^ ^2^ ^]^ and our PLD grown Sr_0.75_NbO₃ with comparable thickness. The transparency enhancement in the UV is likely due to combined effects from Nb, Mo and W with large *E*_O2p‐_ *_B_*_dt2g_. We hypothesize that the IR transparency and the unusually flat IR dispersion observed in transmission and ε_1_ for Sr_0.95_BO_3_ arise from: (1) the increased effective mass resulting from correlation effects and electron‐phonon coupling,^[^ ^33^ ^]^ and (2) the extreme chemical disorder, which suppresses intraband transitions by increasing electron scattering thus reducing low‐energy transition probabilities. To further investigate this, we now examine the electrical properties.

### 4.2 Electrical Properties

**Figure 5:**
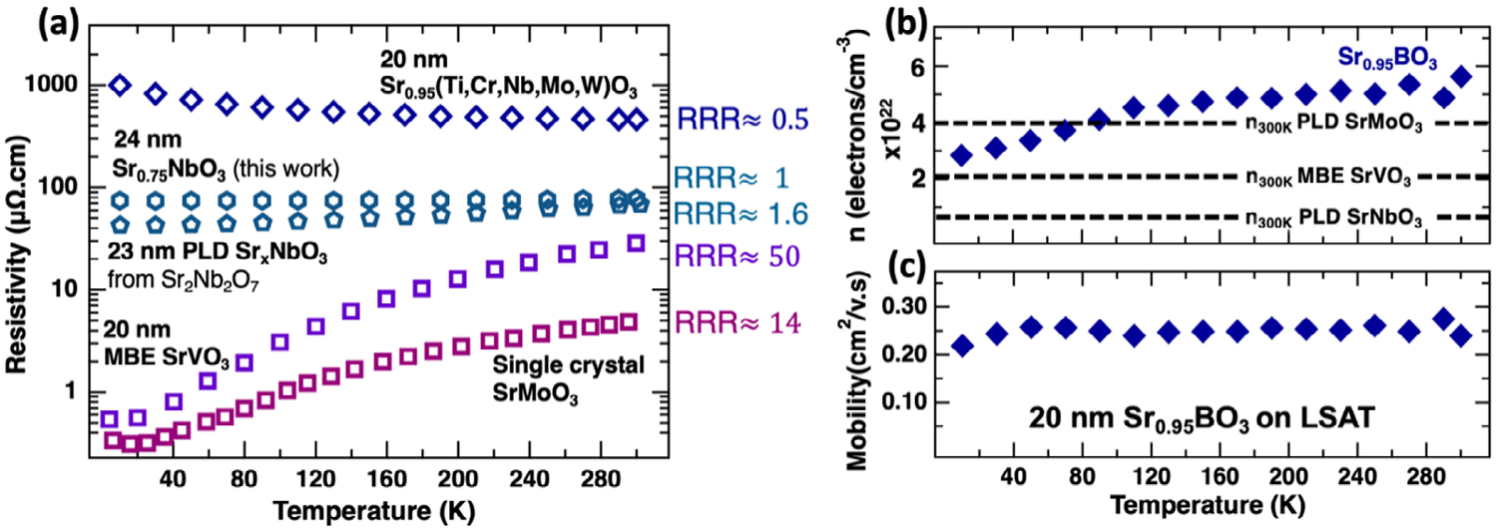
Sr_0.95_BO_3_ electrical properties. a) Temperature‐dependent resistivity for Sr_0.95_BO_3_ compared to that of MBE SrVO_3_^2^, single‐crystal SrMoO_3,_^[^ ^31^ ^]^ PLD Sr_x_NbO_3_ film from Sr_2_Nb_2_O_7_ target,^[^ ^9^ ^]^ and our PLD Sr_0.75_NbO_3_, b) carrier concentration of 20 nm Sr_0.95_BO_3_ film on LSAT as a function of temperature, with values from MBE SrVO_3_^2^, single‐crystal SrMoO_3_^[^ ^31^ ^]^ and PLD Sr_x_NbO_3_ film from Sr_2_Nb_2_O_7_ target^[^ ^9^ ^]^ at 300K plotted for reference, and c) mobility in a 20 nm Sr_0.95_BO_3_ film on LSAT as a function of temperature.

**Figure** **5** shows temperature‐dependent resistivity of Sr_0.95_BO_3_, our PLD‐grown Sr_0.75_NbO_3_, the seminal SrVO_3_ MBE films of Zhang et al.,^[^ ^2^ ^]^ high conductivity PLD Sr_x_NbO_3_ film from Sr_2_Nb_2_O_7_ target by Park et al.,^[^ ^9^ ^]^ and the highest conductivity SrMoO_3_ single crystal from Nagai et al.^[^ ^34^ ^]^ We choose the 20nm Sr_0.95_BO_3_ and the 24nm Sr_0.75_NbO_3_ films for best comparisons to literature reported thicknesses and both films show a weak temperature dependence. In context, all Sr_0.95_BO_3_ end‐members, except band‐insulating SrTiO_3_, are reported or predicted to be weakly metallic.^[^ ^11^, ^12^ ^]^ We attribute the weak Sr_0.95_BO_3_ temperature dependence to the combination of high crystalline fidelity, enhanced electron correlations, and simultaneous chemical disorder, which possibly creates local metallic regions separated by a distribution of small transport barriers that reflect a nonperiodic or pseudoperiodic potential modulated by chemical disorder. Similar behavior is observed at all film thicknesses and on different substrates (Note S15, Supporting Information). To the best of our knowledge, our films exhibit the lowest electrical resistivity reported to date among high‐entropy oxides and retain this record‐low value even at low temperatures.^[^ ^35^, ^37^ ^]^ The 20 nm Sr_0.95_BO_3_ film in Figure 5a reproducibly exhibits a room‐temperature resistivity of ≈460 µΩcm. However, small deviations from the optimized synthesis conditions can increase resistivity by more than 3×, with rapid quenching from the deposition temperature being especially critical. Note S13, Supporting Information details establishing the deposition conditions for achieving high‐conductivity films.

Both mobility and carrier concentration in Sr_0.95_ *B*O_3_ show relatively weak temperature dependence (Figure 5b,c), with mobility remaining nearly flat and carrier concentration decreasing by ≈50% from 300 to 10 K. This modest variation in both quantities contributes to the negative temperature coefficient of resistivity and the small residual resistivity ratio (RRR) relative to end‐members, with the drop in carrier concentration likely dominating the observed resistivity doubling at low temperature. Figure 5b shows that Sr_0.95_ *B*O_3_ has a room temperature carrier concentration ≈5×10^22^ cm^−3^ which is 2× and 4× larger than SrMoO_3_^[13]^ and SrNbO_3,_^[9]^ respectively. We attribute this large value to high band filling from a large overall average *B*‐site valence while maintaining a relatively small unit cell volume – unit cell volume is 1.6% and 4.6% smaller than SrMoO_3_^[13]^ and SrNbO_3,_^[9]^ respectively. Conversely, the 0.22 cm^2^V^−1^s^−1^ room temperature electron mobility in Figure 5c is significantly lower than that of end‐members like SrNbO_3_ (≈8 cm^2^V^−1^s^−1^)_._^[^ ^9^ ^]^ This reduced mobility, like our optical analysis, could be attributed to the increased scattering caused by disorder. Last, we note that the RRR decrease from SrVO_3_ to Sr_0.75_NbO_3_ to Sr_0.95_ *B*O_3_ in Figure 5a follows the trend of increasing chemical disorder, supporting a link between configurational disorder and suppressed temperature‐dependent transport.

## 5 Conclusions, Outlook, and Future Directions

Colloquially, “disorder” connotes chaos, but within crystalline materials it can often be a source of functionality, as in optical glasses, relaxor ferroelectrics, superconductors, and hybrid lead–halide perovskites.^[^ ^38^, ^43^ ^]^ HEOs provide a new landscape to engineer functionality through chemical disorder by preserving long‐range translational order (enabling analyses based on Bloch's theorem) and local connectivity (ensuring consistent coordination geometries).^[^ ^22^, ^25^, ^27^, ^28^ ^]^ Extending the high‐entropy design concept to correlated oxides, we engineer and demonstrate Sr_x_(Ti,Cr,Nb,Mo,W)O_3_ as a pioneering chemically disordered and positionally ordered correlated transparent conductive oxide.

**Figure 6:**
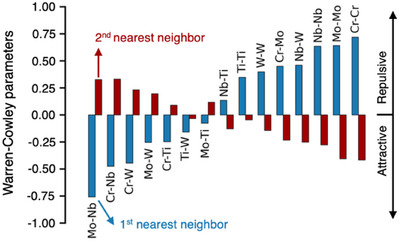
Sr_0.95_BO_3_ short range ordereing. Warren‐Cowley (*w*_αβ,*n*_) parameters for pairs of B‐cations in the first and second nearest neighbor shells equilibrated at 1500K. Positive *w*_αβ,*n*_parameters indicate repulsion; negative *w*_αβ,*n*_ parameters indicate attraction.

A critical next step in understanding far‐from‐equilibrium compositions is uncovering the role of short‐range order and its connection to functional properties. While periodic trends and end‐member DFT calculations (Section 1, Note S2, Supporting Information) provide useful starting points for HEO design, they overlook the chemical inhomogeneity and local interactions that define disordered systems. Accurate prediction of such systems remains a challenge, as theoretical models are still evolving. To move beyond the limitations of 0 K, small‐supercell DFT, we employ a cluster expansion model to probe short‐range ordering at experimentally relevant temperatures – demonstrating a promising yet largely unexplored path toward deeper insights into these complex systems. Specifically, we compute the Warren‐Cowley parameter (*w*_αβ,*n*_) for the first and second nearest neighbor shells in Sr_0.95_ *B*O_3_ equilibrated at 1500 K with Metropolis Monte Carlo sampling, as illustrated in **Figure** **6** (See Note S14, Supporting Information for formal definitions and comment on temperature).^[^ ^44^, ^45^ ^]^ *w*_αβ,*n*_ simply measures the deviation from a random distribution of *α‐β* cation pairs in the *n* ^th^ coordination shell: negative values indicate clustering, while positive values suggest repulsion. The most interesting result from Figure 6 is that Ti‐containing pairs have the smallest *w*_*Ti*β,*n*_ values, suggesting that Ti atoms are adaptable to many local environments and serve as a “mixing” agent. This supports our *Section 1* hypothesis that Ti enhances the stability in the cubic perovskite structure. Additionally, and most generally, cation pairs that are either highly attractive or repulsive in the first shell have the opposite interaction in the second shell, with similar relative magnitude. This suggests an overarching tendency to avoid cation clustering at the polyhedral level, but a possibility for short range ordering at the several‐polyhedral length scale. Both are consistent with the present models that explain the electrical and optical properties. Future work incorporating Sr vacancies and mixed‐valence cations directly into Monte Carlo simulations will further improve our ability to model atomic‐scale interactions and connect them to macroscopic functionality.

Reflecting on the broader implications, electron correlation enriches the complexity of disordered materials, intertwining electrical, optical, and magnetic responses, which make disordered correlated oxides appealing for a wide range of applications from nonlinear transport to quantum applications.^[^ ^7^, ^18^, ^46^ ^]^ Our central result is the ability to stabilize perovskite phases with cations that typically do not form perovskites in their equilibrium ternary forms. Entropic contributions and kinetically arrested synthesis enable this flexibility, allowing compositional tuning far beyond conventional limits. Though originally motivated by UV‐transparent conductors, the implications extend to exotic spin–orbit metals and other emergent functionalities. While a detailed exploration is beyond the scope of this work, we note that successfully incorporating W, with its strong spin–orbit coupling, is a strategic step toward spintronic functionality.^[^ ^7^, ^15^, ^17^ ^]^ Similarly, future targeted B‐site substitutions such as Mn or Fe for ferrimagnetism^[^ ^47^ ^]^ or Cu for superconductivity^[^ ^37^ ^]^ point to a broader composition space ripe for discovery. As shown in Note S15, Supporting Information, the high‐entropy perovskite structure maintains high crystalline fidelity even with substantial compositional changes—including Ti removal, increased W content, Mn addition on the B‐site, and La addition on the A‐site—underscoring its forgiveness and robustness. Overall, this work highlights disorder‐engineered perovskites as a versatile platform for designing new functionalities, with many opportunities still to be explored.

## Conflict of Interest

The authors declare no conflict of interest.

## Author Contributions

S.S.I.A., P.K., R.E.H., J.T.H., and J.P.M. formulated the chemically disordered‐perovskite hypothesis. S.S.I.A. and J.P.M. established the design criteria and overall experimental strategy. S.S.I.A. executed all synthesis, preparing bulk ceramics, depositing thin films, and collecting and analyzing XRD and XPS data; with bulk‐synthesis assistance from M.F. and B.Y. P.K. and J.T.H. led, performed, and interpreted all electrical measurements. J.T.S. and S.B.S. carried out and analyzed the DFT calculations. Optical properties were measured and analyzed by Y.I., S.S., and V.G. S.V.G.A. and N.A. conducted the TEM analysis, while G.B. and C.M.R. obtained and interpreted the XANES data. I.D. and S.G. developed the cluster‐expansion model. S.S.I.A. led the collaborative effort, and led data analysis, visualization, and interpretation, with resources, support and guidance from V.C. and J.P.M. S.S.I.A. and J.P.M. wrote the manuscript, and all authors revised and approved the final version.

## Supporting information



Supporting Information

## Data Availability

The data that support the findings of this study are available in the material of this article.
