# Supplementary material for: Chemically‐Disordered Transparent Conductive Perovskites With High Crystalline Fidelity
Source: Adv Sci (Weinh). 2025 Jul 12;12(42):e09868. doi: 10.1002/advs.202509868 (PMC12622520; doi:10.1002/advs.202509868)
Supplement: Supplementary file 1 — Supporting Information [file ADVS-12-e09868-s001.pdf]

Chemically-Disordered Transparent Conductive Perovskites with High Crystalline Fidelity

*[1]Saeed S. I. Almishal, [2]Pat Kezer, [3]Jacob T. Sivak, [1]Yasuyuki Iwabuchi, [1]Sai Venkata Gayathri Ayyagari, [1]Saugata Sarker, [1]Matthew Furst, [5]Gerald Bejger, [1]Billy Yang, [1]Simon Gelin, [1]Nasim Alem, [1]Ismaila Dabo, [5]Christina M. Rost, [1,3,6]Susan B. Sinnott, [4]Vincent Crespi, [1]Venkatraman Gopalan, [7] Roman Engel-Herbert, [8] John T. Heron, *[1]Jon-Paul Maria

[1]*Department of Materials Science and Engineering, The Pennsylvania State University, University Park, PA 16802, USA*

[2]*Department of Electrical Engineering, University of Michigan, Ann Arbor, MI, 48109, USA*

[3]*Department of Chemistry, The Pennsylvania State University, University Park, PA 16802, USA*

[4]*Department of Physics, The Pennsylvania State University, University Park, PA 16802, USA*

[5]*Department of Materials Science and Engineering, Virginia Polytechnic Institute and State University, Blacksburg, VA 24061, USA*

[6]*Institute for Computational and Data Sciences, The Pennsylvania State University, University Park, PA 16802, USA*

[7]*Paul-Drude Institute for Solid State Electronics, Hausvogteiplatz 5-7, 10117 Berlin, Germany*

[8]*Department of Materials Science and Engineering, University of Michigan, Ann Arbor, MI, 48109, USA*

Corresponding authors: Saeed S. I. Almishal saeedsialmishal@gmail.com and Jon-Paul Maria jpm133@psu.edu

Keywords: disorder, electron correlation, high-entropy oxides, thin films, perovskites, transparent conductors, XRD, XPS, TEM, DFT, cluster expansion

Abstract

This manuscript presents a working model linking chemical disorder and transport properties in correlated-electron perovskites with high-entropy formulations and a framework to actively design them. We demonstrate this new learning in epitaxial $Sr_x(Ti,Cr,Nb,Mo,W)O_3$ thin films that exhibit exceptional crystalline fidelity despite a diverse chemical formulation where most *B*-site species are highly misfit with respect to valence and radius. X-ray diffraction, X-ray photoelectron spectroscopy, and transmission electron microscopy confirm a unique combination of chemical disorder and structural perfection in thin and thick epitaxial layers. This combination produces an optical transparency window that surpasses that of the constituent end-members in the UV and IR, while maintaining relatively low electrical resistivity. We address the computational challenges of modeling such systems and investigate short-range ordering using cluster expansion. These results showcase that unusual *d*-metal combinations access an expanded property design space that is predictable using end-member characteristics and their interactions – though unavailable to them – thus offering performance advances in optical, high-frequencey, spintronic, and quantum devices.

Methods and Supplementary Information

Experimental Methods

Sample Synthesis Sr_xBO_3 were deposited on different substrates by PLD using a Coherent 248 nm KrF laser ablating sintered ceramic targets (supplementary note 5). Substrates were cleaned with methanol followed by a 1hr- UV-ozone treatment then were adhered to a heater buck with silver paint (Ted Pella Leitsilber 200). The substrates were transferred to the heater at 850°C and growth started 3 minutes after transfer. 40 sccm Argon was flowed into the chamber, the gate valve was throttled until the chamber pressure stabilized at 50mTorr. Laser fluence was 1.4 J/cm^2 with a total laser pulse energy of 110 mJ. All films were quenched to ambient lab conditions in less than 6 minutes after the final laser pulse to mitigate post-synthesis annealing effects (See supplementary Note 13 for more details).

X-ray diffraction (XRD) was performed using a PANalytical Empyrean diffractometer with Bragg-Brentano HD (BBHD) incident beam optics. We employed a 2-bounce Ge hybrid monochromator on the incident side for the high-resolution scans and reciprocal space maps (RSMs). The primary diffracted beam optic was a programmable divergent slit and a PIXcel^{3D} detector combination. For high resolution wide XRD scans, Figure 2(b), we used a 2-bounce Ge crytal analyzer on the detector side with a proportional detector.

X-ray photoelectron spectroscopy (XPS) experiments were performed using a Physical Electronics VersaProbe III instrument equipped with a monochromatic Al kα x-ray source (hν = 1,486.6 eV) and a concentric hemispherical analyzer. Charge neutralization was performed using both low energy electrons (<5 eV) and argon ions. The binding energy axis was calibrated using sputter cleaned Cu (Cu $2p_{3/2}$ = 932.62 eV, Cu $3p_{3/2}$ = 75.1 eV) and Au foils (Au $4f_{7/2}$ = 83.96 eV). Peaks were charge referenced to CH_x band in the carbon 1s spectra at 284.8 eV. In cases where additional correction was necessary to account for potential screening effects, we used Sr $3d_{5/2}$ peak at 132.9 eV as correction reference. Measurements were made at a takeoff angle of 85° with respect to the sample surface plane. This resulted in a sampling depth of roughly 4.5-9.5 nm (95% of the signal originated from this depth or shallower). Quantification was done using instrumental relative sensitivity factors (RSFs) that account for the x-ray cross section and inelastic mean free path of the electrons. On homogeneous samples major elements (>5 atom%) tend to have standard deviations of < 3% while minor elements can be significantly higher. The analysis size was ~200μm in diameter. All fittings were performed in CasaXPS. More details are available in Supporting Information Note 8.

Transmission Electron Microscope Sample preparation for S/TEM studies were carried out using Scios 2 Focused Ion Beam (FIB). The cross-sectional specimen was extracted at 30kV ion beam and thinned at 5kV ion beam. Finally, 2kV ion beam was used to clean the sample. Selected Area Electron Diffraction experiments were performed on Talos X2 at 200 kV accelerating voltage. The selected area aperture was placed over both the film and substrate. STEM and STEM-EDX studies were carried out at a 300 kV accelerating voltage on the aberration-corrected Titan G2 microscope at MCL, Penn State. Drift correction on HAADF-STEM image was performed using open-source

MATLAB code on images acquired in orthogonal scan directions.[1]

Ellipsometry spectra in (Ψ,Δ) were collected using three different ellipsometry. J.A.Woollam M-2000 Ellipsometer was used at the incident angle of 45° to 85° for the spectral range of 0.734 to 5.042 eV. J.A.Woollam M-2000F Focused Beam Ellipsometer was used at the incident angle of 64.54° for the spectral range of 1.240 to 6.458 eV. Prior to the measurement of SrBO3 samples, the ellipsometry spectra of LSAT substrate were measured for the modeling. The data were modeled by B-spline fitting with Kramers-Kronig relations using J.A. Woollam CompleteEASE software.

Ultraviolet-Visible-near-IR Spectroscopy The transmission (%) and reflection (%) measurements in the wavelength range from 250 to 2500 nm (4.959 to 0.4959 eV) were measured using an Agilent Cary5000 Ultraviolet-Visible-near-IR Spectroscopy with an integral sphere. The transmission intensity of a sample was normalized by the intensity without a sample in the same optical path. The reflection intensity of a sample was normalized by the intensity of the Spectralon, the reflectance standard.

Fourier-Transform Infrared (FT-IR) Spectroscopy The transmission (%) and reflection (%) measurements in the wavelength range from 2500 to 16000 nm (0.4959 to 0.07749 eV) were performed using a Bruker Hyperion 3000 Microscope with a 15× objective lens. The transmission intensity of a sample was normalized by the intensity without a sample in the same optical path. The reflection intensity of a sample was normalized by the intensity of the gold film on the Si substrate.

Temperature dependent Hall and resistivity measurements were performed in a Physical Properties Measurement System (PPMS). The samples were configured in the standard Van der Pauw geometry. A baffle rod fixed with a Au-coated sealing disc hovered <1 cm above the sample to ensure thermal equilibration; additionally, the sample was held at each temperature for the same purpose. The thermal rate between measurements was set to 5 K/min. Sheet resistance and Hall effect measurements were performed using a Keithley 2450 Source Measure Unit and a Keithley 3706A-S to switch amongst all to Van der Pauw configurations. The source current ranged from 100 to 200 μA. The magnetic field was swept between ±2 T for Hall voltage measurements on LSAT. We do not report the Hall measurements as a function of temperature for the films on GSO and DSO due to their strong paramagnetic background. For carrier concentration measurements, Hall effect data may overestimate the number of free carriers due to contributions from hopping transport, which can also generate a Hall voltage. While these values should be interpreted with caution, they nonetheless provide a useful basis for comparison with previously reported values in the literature obtained using similar measurement techniques.

Supplementary Notes

Note 1: Clarifying Terminology and Thermodynamic Principles

Chemical disorder, high entropy and entropy stabilized

In this study, we utilize the term "High-Entropy Oxide" (HEO) to describe crystalline oxide solid solutions characterized by four- or five-component near-equimolar cation mixtures occupying one or more equivalent lattice sites. This yields a large configurational mixing entropy, S_{config}. From a crystallographic viewpoint, a loosely defined crystal lattice still exists on average, with an indeterminate identity atom occupying each lattice site. We use the term "end-members" in reference to the constituent extremes of the compositional range in HEO solid solutions – each endmember has one determinate atom occupying equivalent lattice sites[2].

The terms "entropy" and "disorder" are often used interchangeably in the literature, though there is a subtle difference between them. "Disorder" implies a focus on a single configuration, whereas "entropy" encompasses a class of configurations. When describing HEOs as disordered, we refer to the inherent local asymmetries within the crystal in a "trapped" state which give rise to their unique properties and functionality. Simultaneously, the significant entropy gain from chemical mixing facilitates HEO phase formation, as the large S_{config} dominates the entropy of mixing (Δs) and boosts the chemical potential change ($\Delta\mu = \Delta h - T\Delta s$) associated with forming a multi-component solution from the end-members, h is molar enthalpy and T is temperature. "Entropy-stabilized" is a more specific term introduced by Rost et. al[3] that implies that $\Delta h > 0$ and that a critical thermodynamically determined temperature exists, above which entropy drives phase formation by making the overall $\Delta\mu$ negative. We consider the main composition in this work to be a structurally ordered, chemically disordered high-entropy oxide, stabilized in part by configurational entropy and kinetically trapped through the rapid quenching enabled by pulsed laser deposition.

Pulsed Laser Deposition (PLD) and Kinetic Stabilization

Pulsed laser deposition (PLD) often employs non-equilibrium kinetics and involves condensing precursors from a high-entropy initial state, allowing kinetic stabilization of a broader spectrum of atomic and electronic configurations whose bulk synthesis may require extreme physical or chemical conditions. The high-entropy phase is subsequently trapped at room temperature with unique local asymmetries and fluctuations in chemical, structural, and electromagnetic order parameters. For example, we have shown in previous studies that controlling PLD growth conditions allows for controlling cations' oxidation states and for subtle nanoscale microstructural reconfiguration[4,5].

Correlation and disorder

To better appreciate correlation's role under disorder, we examine two extreme hypothetical cases from the electrons' perspective: one with metallic components exhibiting little to no electron correlation, and the other with insulating oxides where electrons are fully localized in closed electronic shells. In the former case, free electrons will screen chemical disorder, minimizing its

impact on transport properties apart from a likely reduction in electronic conductivity. In the latter case, disorder may primarily influence the optical behavior. By permitting oxygen vacancies and cation multivalency, however, disorder can introduce unique magnetic phases, electrochemical activity, ionic conductivity, and defect-mediated hopping conduction in insulating oxides.[6–11]

The intermediary case of correlated electron end-members, as presented in the main manuscrpt, therefore offers an ideal setting for investigating and engineering complex electron behaviors and interactions. Disorder in these systems amplifies correlated electron interactions, intertwining electrical, optical, and magnetic responses. This fuels interest in disordered correlated oxides for developing various quantum applications and devices, even beyond conventional linear transport engineering including disorder induced nonlinear and high-order hall effects (See Supplementary Note 15 for more discussion).[12–14]

Note 2: DFT calculations

Magnetic ordering and symmetry breaking surpass naïve DFT in capturing correlation

While long-range symmetry provides a starting point for modeling electron-correlated HEOs, the enormous number of atoms necessary to accurately model these disordered systems as well as the lack of analytical solutions to fully account for correlation poses significant challenges to our theoretical and computational predictability. Nonetheless, straightforward DFT calculations coupled with periodic trends offer a wealth of crystal chemistry rules to inform our composition selection, as outlined in Section 1in the main text. Despite being common in literature, the so-called "naïve" DFT that utilizes only the cubic, nonmagnetic (NM) unit cell for perovskites omits and underestimates correlation compared to the more realistic, paramagnetic (PM) material, as has been recently shown for a variety of different perovskites[43]. Allowing colinear spins in an anti-ferromagnetic ordering (AFM-G) for 2x2x2 supercells in which all spins have maximum spin dissimilarity, however, results in an overestimate of correlation[15,16] . While neither method fully encapsulates the true PM behavior at room temperature, we consider both NM and AFM-G configurations in this study to provide a more comprehensive computational view for the explored $SrBO_3$ end members. We find that this provides valuable insights for designing and understanding our champion composition. The Vienna Ab-initio Software Package (VASP) 6.4.1 is used for DFT calculations with the projector augmented wave pseudopotentials v54.[17] The regularized-restored strongly constrained and appropriately normed (r^2SCAN) functional is used for its improved accuracy, hence here we did not consider the use of Hubbard U corrections.[18] Optical calculations are performed using the independent particle approximation for calculating the frequency-dependent dielectric matrix, in which the number of bands was doubled to ensure convergence of the energy spectrum. A Γ-centered k-point mesh of 8x8x8 was used for the 5-atom unit cells and scaled linearly with the size of the supercell. The k-point mesh was tripled (i.e. Γ-centered 24x24x24 for the unit cell) for band structure and optical calculations. To allow for symmetry breaking distortions (such as octahedral tilting), we rattle all atoms prior to starting the relaxation process; forces were minimized to less than 10 meV/Å, and the global cubic symmetry was maintained. Pymatgen[19], the Atomic Simulation Environment[20], and SUMO[21] were used for

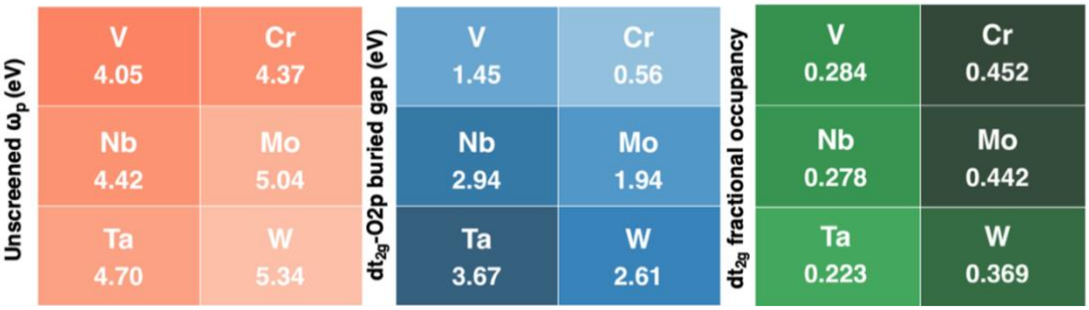

Figure S1. DFT nonmagnetic calculations heat maps for unscreened plasma frequency, d_{2g}-O_{2p} buried gap and dt_{2g} fractional occupancy.

analysis. The unscreened plasma frequency was extracted from calculated frequency dependent dielectric matrices calculated using the LOPTICS = True flag in VASP while $E_{O2p\text{-}t2g}$ gaps were determined using the atom-projected density of states. For completeness, we include in Figure S1 the heat maps for the NM calculations for Figures 1(c), 1(e), and 1(g). Figure S2 and Figure S3 present a complete set of NM and AFM-G band structures for the relevant end members - providing a clear comparison between the two methods. Notably, different than NM calculations, for AFM-G calculations we utilize large supercells that allow for structural and magnetic symmetry-breaking motifs that have shown to be important in accurately predicting these quantities as outlined below. These supercells calculations, however, has the disadvantage of nonintuitive and dense band structures[16]. To overcome this, we apply band unfolding to the supercell band structure, resulting in an effective band structure with spectral functions rather than the sharp bands found in the unit cell, as demonstyrated in Figure S3. Band unfolding is performed with easyunfold software[22].We also utilize these supercells with AFM-G magnetic ordering for binary B-site perovskite Bader charges calculations. Bader charges were calculated with the software from the Henkelman group[23].

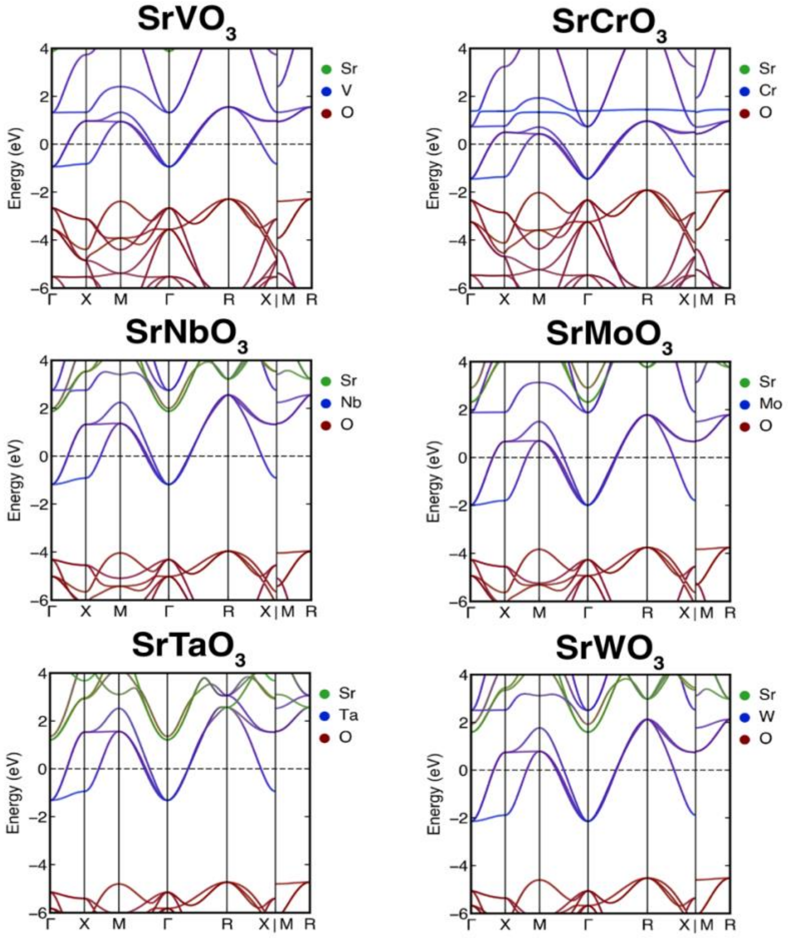

Figure S2. $SrBO_3$ NM end-member band structures

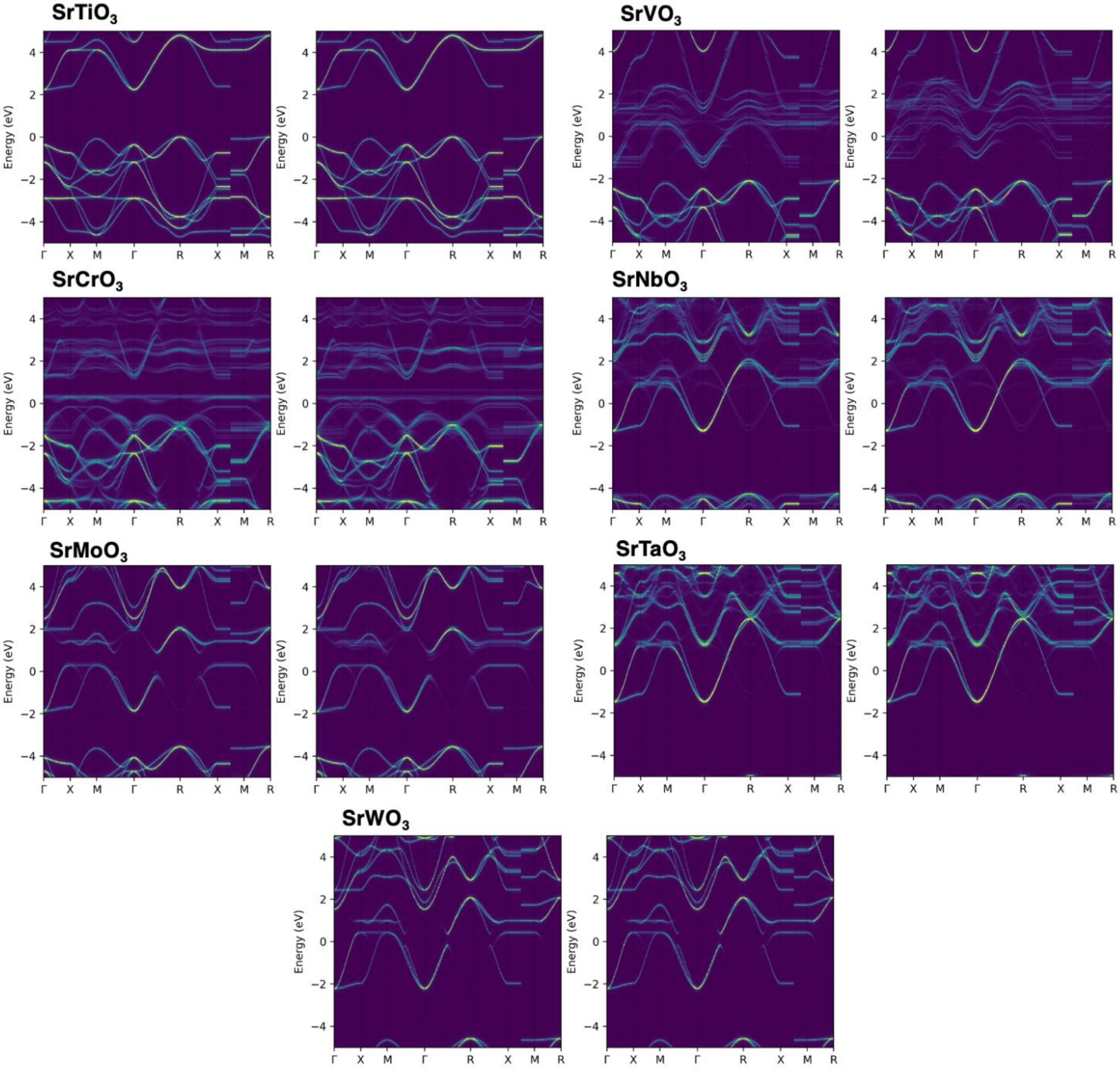

Figure S3. $SrBO_3$ AFM-G end-member band structures Brighter colors indicate higher density of electronic states, with spin up and down on the left and right, respectively.

DFT endmembers optical absorption

Figure S4 presents the optical absorption spectra of all relevant end-members, calculated from DFT using NM and AFM-G ordering. Note that AFM-G results in higher absorption features in the visible and ealy UV regimes compared to NM calculations. Inspecting the absorption sprectra calculated with AFM-G ordering, $SrNbO_3$ and $SrTaO_3$ exhibit the lowest absorption in the UV and visible ranges, while $SrTiO_3$ shows the lowest absorption in the visible and infrared ranges. $SrVO_3$ and $SrCrO_3$ demonstrate better transparency in the visible range compared to $SrMoO_3$ and $SrWO_3$, and they outperform all other early transition metals from groups 5 and 6 in the lower energy ranges.

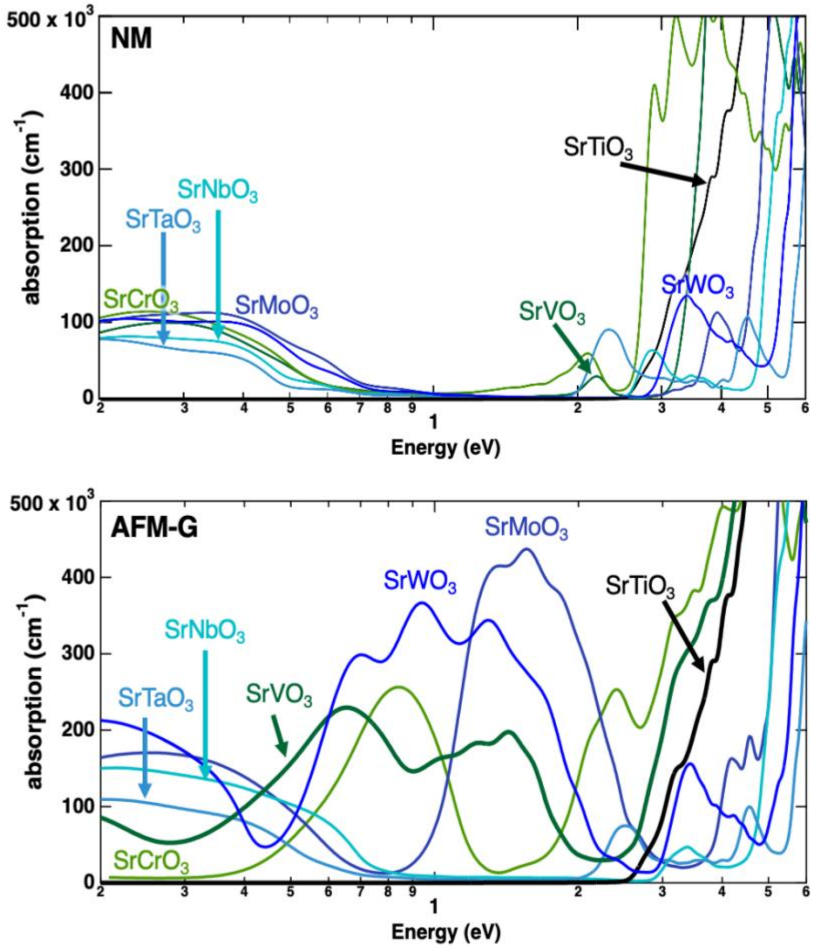

Figure S4. NM vs AFM-G $SrBO_3$ end-members optical absorption

Bader charge change in DFT binary B-site cations

Bader charge analysis of binary B-site perovskites $Sr(B^1B^2)O_3$, presented in Figure S5, crudely captures first-neighbor cation interactions—unlike single-element 4+ B-site calculations, and reveals significant deviations from the nominal 4+ oxidation state, indicating tendencies toward further oxidation or reduction. The cations are ordered from left to right according to their predicted likelihood of forming first nearest-neighbor pairs, as determined by the cluster expansion model discussed in Figure 6 of the main text and detailed further in Supporting Information Note 14. $Sr(B^1B^1)O_3$ compositions are included as placeholders for reference. The central insight from this analysis is that cations actively shift their oxidation states away from the nominal 4+ to achieve energetically favorable configurations. For instance, as discussed in the main text, Cr strongly favors the 3+ oxidation state. This reduction in Cr is balanced by further oxidation of neighboring cations such as Nb, Mo, and W. This extensive diversity of cations and their accessible oxidation states constitutes what we refer to as the **valence milieu**.

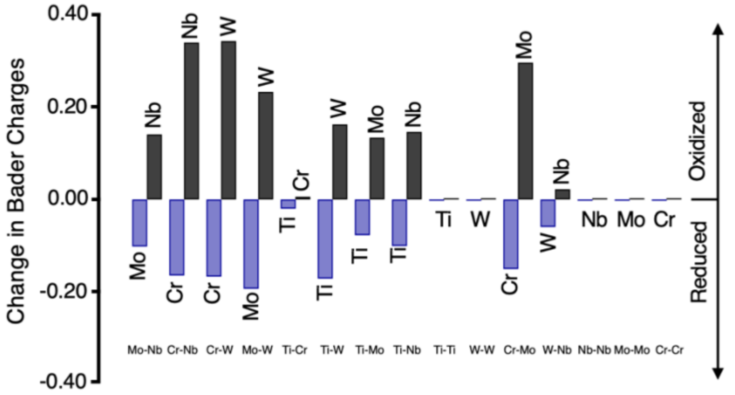

Figure S5. DFT AFM-G calculations for the change in bader charges in binary B-site perovskites; $Sr(B^1B^2)O_3$

Note 3: Preparing the ceramic target

Bulk ceramics were prepared by mixing binary oxide powders to give stoichiometric solid solutions, namely SrO, TiO_2, Cr_2O_3, Nb_2O_5, MoO_3 and WO_3 purchased from Millipore-Sigma. Powders were checked for phase and stoichiometry at every processing step using X-ray diffraction (as discussed in Methods) and PANalytical Epsilon 1 X-ray fluorescence. We reacted the B-cations first at 650°C for 24 hours to avoid MoO_3 sublimation and to ensure proper B-site cations mixing. Then we added SrO to the B-cations mix at ambient conditions and shaker-milled the mix with 5 mm and 3mm diameter yttrium-stabilized zirconia milling media for 18 hrs. Subsequently, we reacted the mix at 1200°C for 24 hours to form Sr_xBO_δ. Then we milled the reacted powder with 5 mm and 3mm diameter yttrium-stabilized zirconia milling media for another 18 hrs. Following that, we pressed the powder uniaxially into a 1" diameter pellet at 140 MPa (Carver Laboratory Press). The target pellet was then sintered in air at 1400°C for 18 hrs and air-quenched by direct extraction from the hot zone of the furnace. The XRD and SEM EDX of the bulk ceramic with 5% Sr vacancies are shown in Figure S6. The target exhibits two phases: one reminiscent of the perovskite structure and the other of the scheelite structure. From EDX maps, Ti, Cr and Nb seem to cluster in the same grains while Mo is well dispersed in the sample.

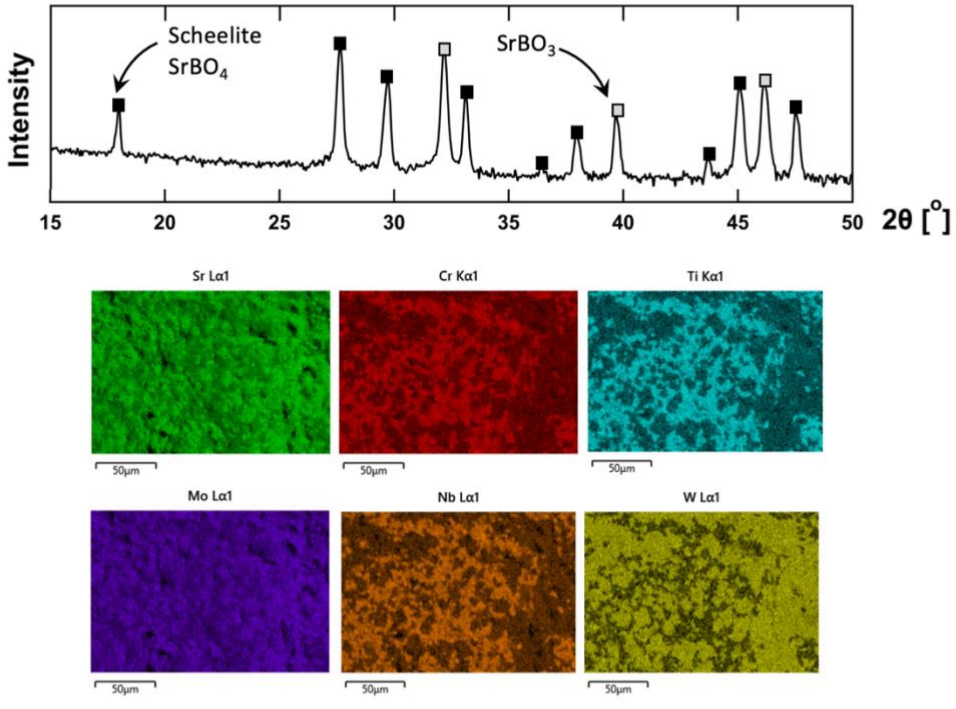

Figure S6. $Sr_{0.95}BO_3$ ceramic target powder diffraction and EDX maps

Note 4: Vacancies on the A-site

Inspired by previous work on Sr_xNbO_3[24], we investigated introducing Sr-vacancies on the A-site when preparing the PLD targets. Among those in Figure S7, films grown from $Sr_{0.95}BO_3$ consistently have the highest crystalline quality and lowest electrical resistivity. Future systematic experiments are needed to elucidate the role that vacancies play in the overall behavior of this perovskite family.

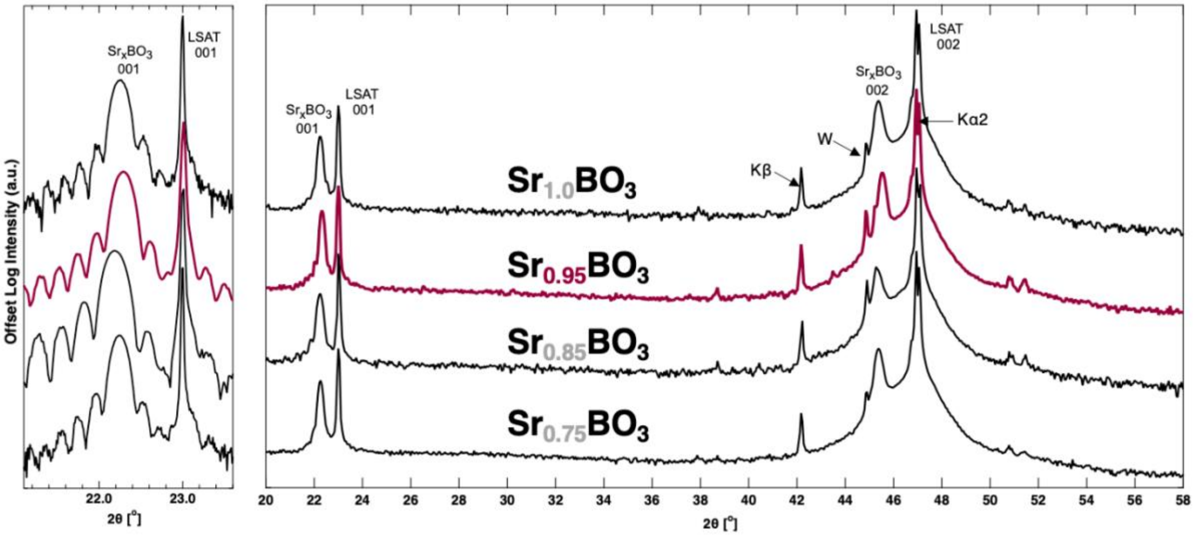

Figure S7. BBHD and high-resolution X-ray diffraction patters for films grown from a series of targets with varying A-site vacancies

Note 5: Growth on different commercially available substrates

Figure S8 depicts wide-angle 2θ−ω XRD scans for $Sr_{0.95}BO_3$ films grown on different technologically relevant substrates: (001) $(LaAlO_3)_{0.3}(Sr_2TaAlO_6)_{0.7}$ (LSAT), (001) $SrTiO_3$, (110) $DyScO_3$, (110) $GdScO_3$ and (001) $KTaO_3$, corresponding to Figure 2a. The surface morphology of

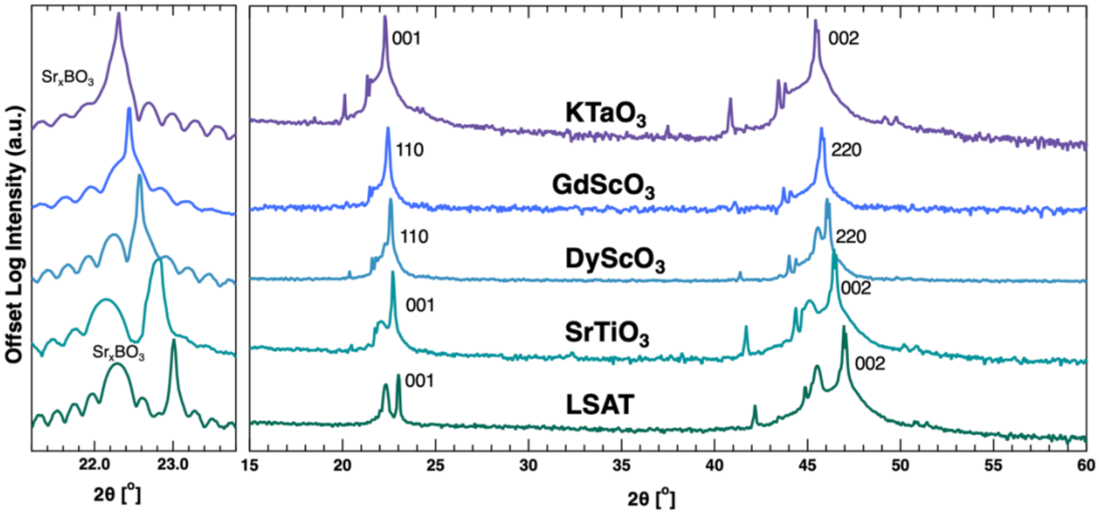

Figure S8. BBHD and high-resolution X-ray diffraction patters for $Sr_{0.95}BO_3$ 35 nm films grown on different substrates

the same set of films was characterized using an Asylum MFP3D atomic force microscope (AFM) in tapping mode as shown in Figure S9. AFM images indicate comparable smooth surfaces on all relevant substrates in this study. The temperature dependent resistivity for another set of films with 20 nm thickness are reported in Figure S10. The 20 nm films were chosen to facilitate comparison with existing literature (see Supplementary Note 13).

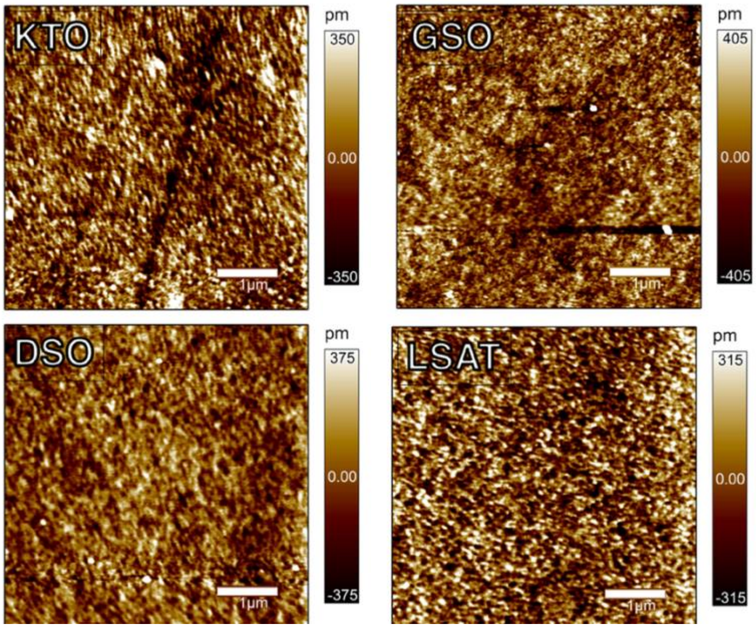

Figure S9. $Sr_{0.95}BO_3$ 35nm films AFM images

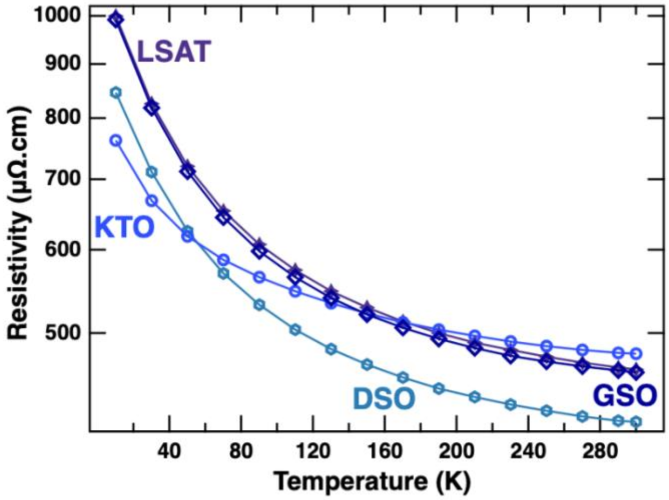

Figure S10. Temperature dependent resistivity of $Sr_{0.95}BO_3$ 20 nm films grown on different substrates

Note 6: Thickness Series on LSAT

Even though the films have the highest lattice mismatch with LSAT compared to other substrates, LSAT substrates are economic, are non-magnetic at low temperatures, have inherently large ultraviolet transmittance, remain insulating and do not react with the films. These characteristics make them suitable for measuring the films' electronic, optical, and magnetic properties with minimal disturbances. Therefore, we highlight $Sr_{0.95}BO_3$ thickness series grown on LSAT. Figure S11 presents wide 2θ- ω scans up to 110° using BBHD optics and the corresponding rocking curves (ω scans at constant 2θ) for the films presented in Figure 2b. Only peaks associated with 001 perovskite phase are present. The ω scans consist of a two-component superposition, one with a full-width-at-half-maxima (FWHM) of (0.18-0.37)° and the other with a FWHM of (0.028-0.045)°. This narrow spread in ω indicate very high-crystalline fidelity particularly in thicker samples. Furthermore, we sustain this high quality up to 300nm thickness as shown in Figure S12.

The TEM analysis in this study was performed on a 35 nm $Sr_{0.95}BO_3$ film on LSAT. Figure S13(a) represents the selected area corresponding to the electron diffraction in Figure 2d. The electron diffraction was acquired from both the HEO thin film and the LSAT substrate, and the lattice mismatch between LSAT and HEO is clearly observed in the diffraction pattern. Figure S13(b) presents an Annular Bright Field STEM image that highlights the positions of both oxygen atoms and cations, while Figure S13(c) shows the simultaneously acquired HAADF STEM image. Both STEM images illustrate a pristine cubic perovskite pattern. Bandpass filtering was applied to the atomic resolution STEM images in Figure S13 using Digital Micrograph. Figure S14 presents the EDX maps for each individual element, deconvoluting Figure 2f.

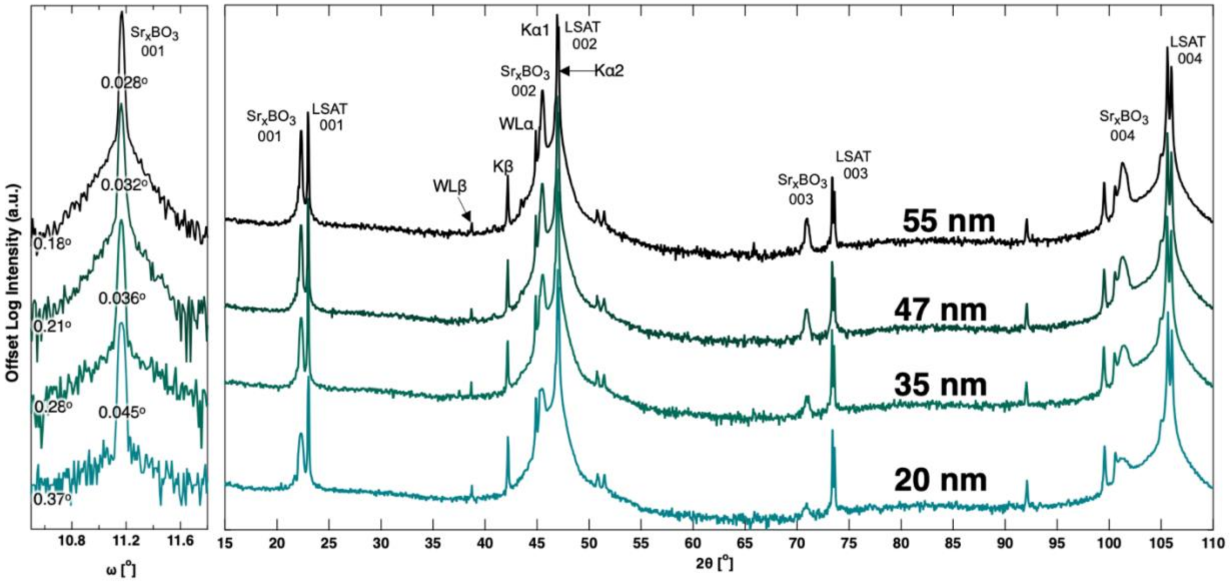

Figure S11. BBHD X-ray diffraction patterns and rocking omega curves for the $Sr_{0.95}BO_3$ films grown on LSAT from 20 nm to 55nm and shown in Figure 2 in the manuscript

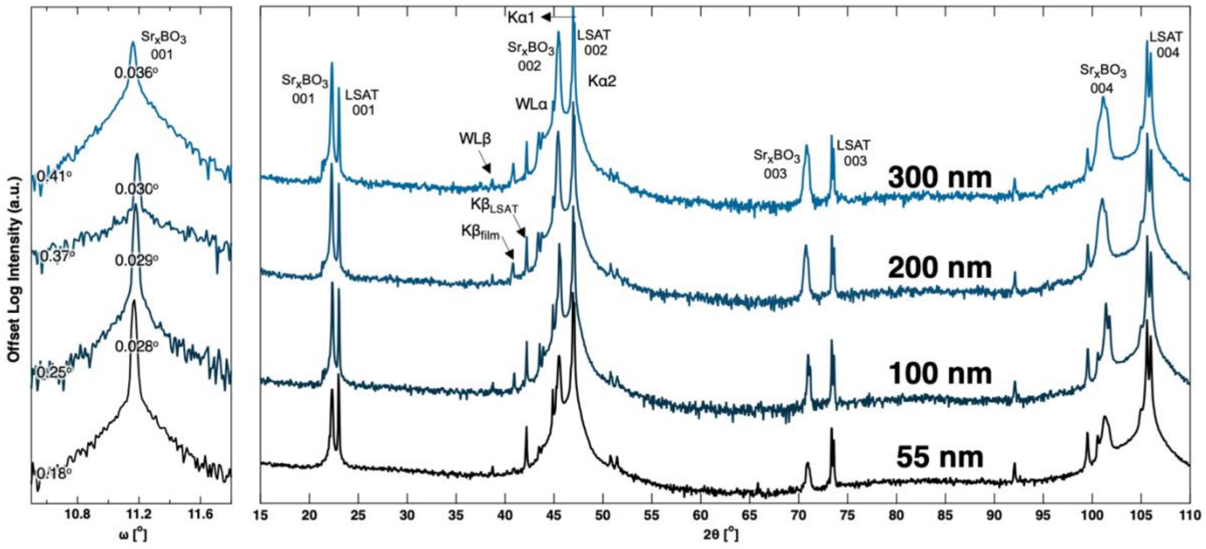

Figure S12. BBHD X-ray diffraction patterns and rocking omega curves for a series of $Sr_{0.95}BO_3$ thin films grown thick up to 300 nm

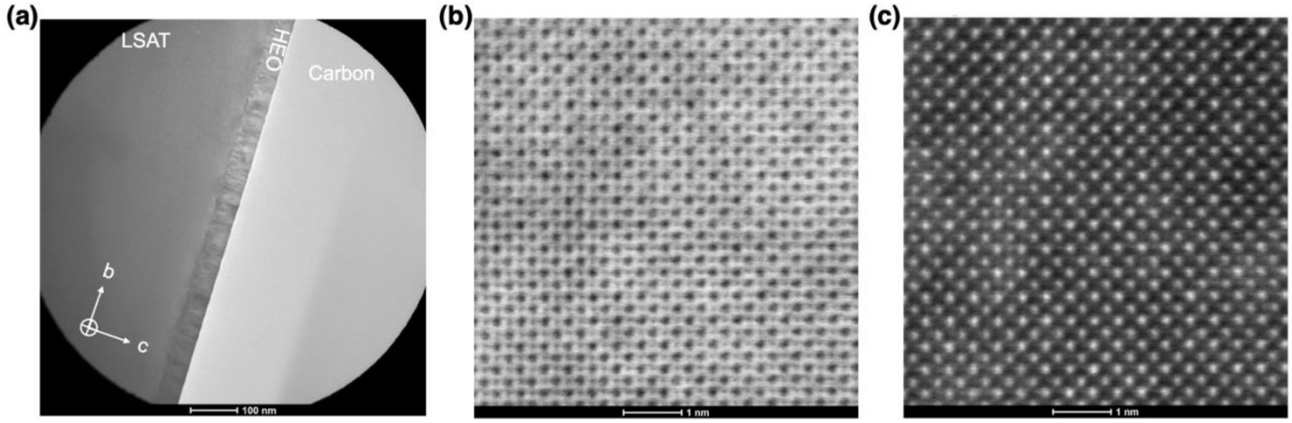

Figure S13. (a) the selected area corresponding to the electron diffraction in Figure 2d, (b) an Annular Bright Field STEM image that highlights the positions of both oxygen atoms and cations, while (c) shows the simultaneously acquired HAADF STEM image.

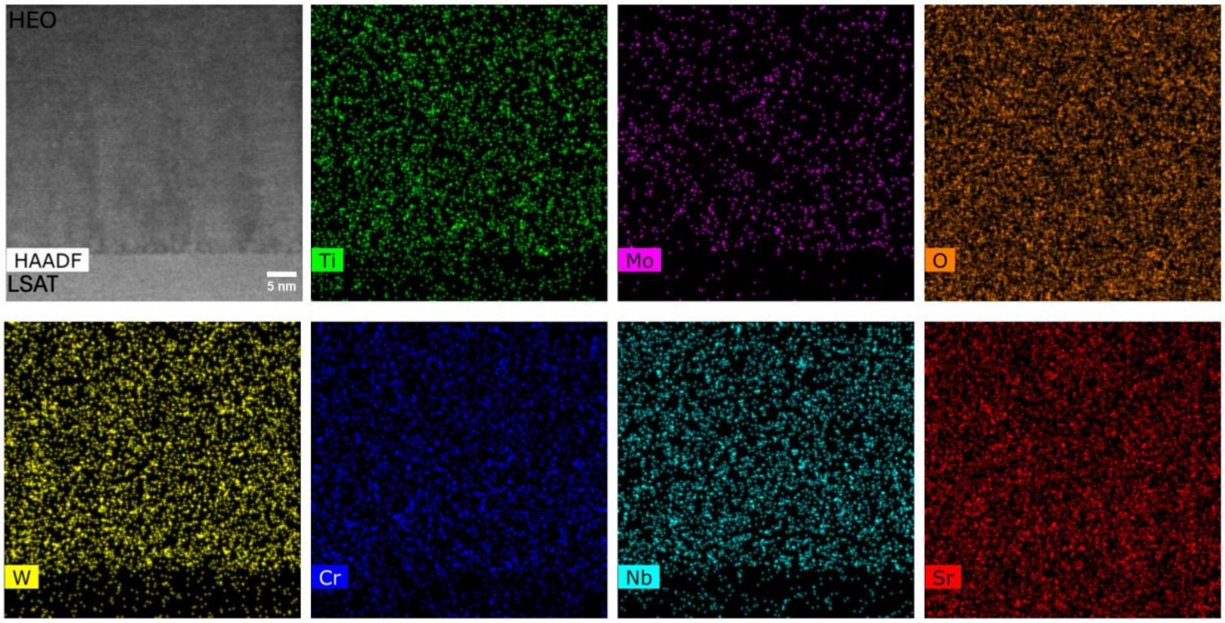

Figure S14. EDX maps of all individual elements in $Sr_{0.95}BO_3$ and the corresponding HAADF image

Note 7: Second harmonic generation and "global" centrosymmetry

We conducted an optical second harmonic generation (SHG) experiment using an 800 nm fundamental laser (Spectra-Physics Ti: sapphire gain medium, 80 fs, 1 kHz) at 45° oblique incidence reflection geometry. The setup is shown in Figure S15. The laser was focused with a 10 cm convex lens, achieving a beam diameter of 25 μm at the focus, measured using a knife-edge technique. The laser-induced surface damage threshold was determined to be 225 GW/cm² by exposing the thin film to various fluences and inspecting for damage under an optical microscope. Nonlinear second harmonic intensity was then measured as a function of the incident intensity.

SHG is a nonlinear optical process where a material generates polarization at frequency 2ω from an incident ω beam, described by second-order optical susceptibility, $P_i^{2\omega} = d_{ijk}E_j^{\omega}E_k^{\omega}$.[25] In a non-centrosymmetric material, the intensity dependence of the second harmonic should be quadratic relative to the incident beam ($I^{2\omega} = \mathrm{A}^*(I^{\omega})^2$). Measurements from this experiment showed that the generated 2ω beam intensity did not follow a quadratic dependence expected of a non-centrosymmetric material (Figure S15), indicating that $Sr_{0.95}BO_3$ films on LSAT are centrosymmetric within the 25 μm probe region.

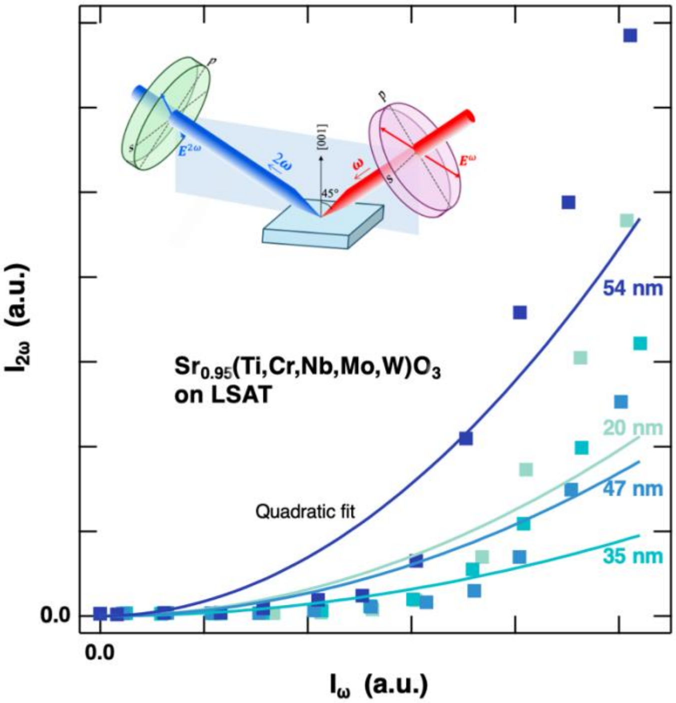

Figure S15. SHG measurements showing the intensity of generated second harmonic response at frequency 2ω as a function of incident laser intensity at the fundamental frequency ω for $Sr_{0.95}BO_3$ films on LSAT shown in Figure 2.

Note 8: $Sr_{0.95}BO_3$ XPS data analysis

Figure S16 depicts the XPS spectra for Sr 3d, Ti 2p, and Cr 2p3/2. The spectra confirm that Sr predominantly adopts the 2+ oxidation state, Ti primarily exists in the 4+ oxidation state, and Cr is mainly present in the 3+ oxidation state. A summary of all observed oxidation states from XPS analysis is provided in Table S1. The fitting process for Nb, Mo, and W is more complex, with the final results of the fitting detailed in the main text (Figure 3). We performed the Nb fitting using $SrNbO_3$ as a reference, as described in Note 10. For Mo and W, we utilized an available set of standard reference data alongside line shapes we derived from fitting of MoO_3, MoO_2, MoS_2, $SrMoO_3$ and WO_3 to fit the $Sr_{0.95}BO_3$ peaks accurately. We use a Lorentzian (LF) line shape with tail damping to fit all peaks, except for the Mo^{4+} peaks, for which we applied a Lorentzian Asymmetric (LA) line shape to account for the asymmetric features characteristic of metallic core levels. To ensure reliability, XPS data fitting was tested for self-consistency across 30 $Sr_{0.95}BO_3$ samples. Additionally, the entire valence determination experiment was validated by applying charge neutrality, as detailed in Table S2, using the same sample analyzed in the EDS mapping in Figure S14. The final result demonstrates that the summation of positive and negative charges—accounting for all measured valence states and the intentional Sr vacancy concentration—is equal and opposite, satisfying charge neutrality.

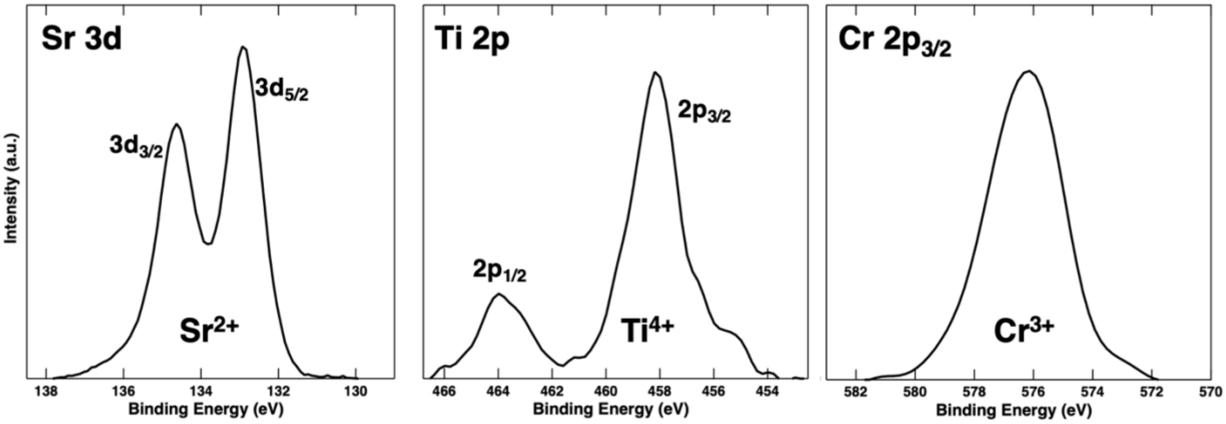

Figure S16. XPS spectra of Sr, Ti and Cr in 35 nm $Sr_{0.95}BO_3$

Table S1: $Sr_{0.95}(Ti,Cr,Nb,Mo,W)O_3$ cation oxidation states observed in XPS

Cation	Oxidation states observed in XPS
**Sr**	2+
**Ti**	4+
**Cr**	3+
**Nb**	2+, 4+ and 5+ (possibly 3+)
**Mo**	4+, 5+ and 6+
**W**	4+ and 6+

Table S2: $Sr_x(Ti,Cr,Nb,Mo,W)O_{3+d}$ charge neutrality calculations

element	stoichiometry	average oxidation per element	total valence
**Sr**	0.92	2	1.84
**Ti**	0.18	4	0.72
**Cr**	0.19	3	0.57
**Nb**	0.21	3.97	0.8337
**Mo**	0.2	4.94	0.988
**W**	0.21	5.54	1.1634
**O**	3.058	-2	-6.116
		**sum**	-0.0009

Note 9: $SrNbO_3$ growth and optical properties from Sr_xNbO_3 targets

Similar to Note 4, we grew Sr_xNbO_3 thin film series using PLD from targets prepared with x = 0.5, 0.75 and 1. Films grown from $Sr_{0.75}NbO_3$ target, exhibit the highest crystalline quality (Figure S17) and best optical performance while maintaining low extinction coefficient k (Figure S18) and high electrical conductivity of 300-400 µOhm.cm at room temperature. Therefore, we choose the film grown from $Sr_{0.75}NbO_3$ target in Figure S17 for the optical comparison with the high entropy system in Figure 4.

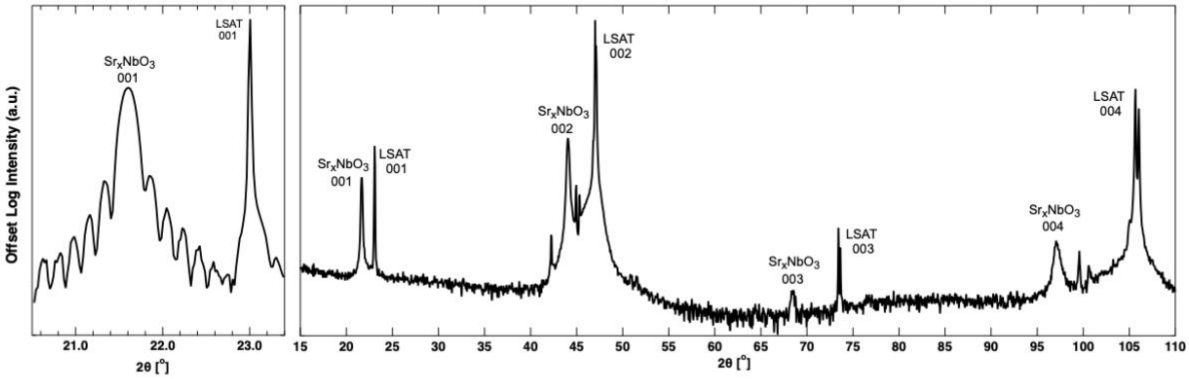

Figure S17. BBHD and high-resolution X-ray diffraction scan of Sr_xNbO_3 film grown on LSAT from $Sr_{0.75}NbO_3$ target

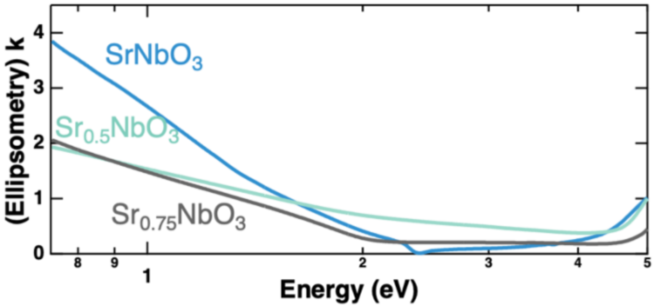

Figure S18. Extinction coefficient of three films grown from $SrNbO_3$, $Sr_{0.75}NbO_3$ and $Sr_{0.5}NbO_3$ ceramic targets, respectively.

Note 10: Nb^{2+} evolution in Sr_xNbO_3 X-ray photoelectron spectroscopy

To achieve a proper fit of the Nb 3d spectra, we followed the approach outlined by Roth et al.[24], incorporating contributions from Nb^{3+} and Nb^{2+} states. As shown in Figure S19, the Nb^{2+} concentration increases with rising Sr vacancies on the A-site in Sr_xNbO_3. We applied the same fitting model as a starting point for Nb in $Sr_{0.95}BO_3$. However, for the high-entropy oxide samples, adding Nb^{3+} did not significantly enhance the fit, so we excluded it to avoid overfitting.

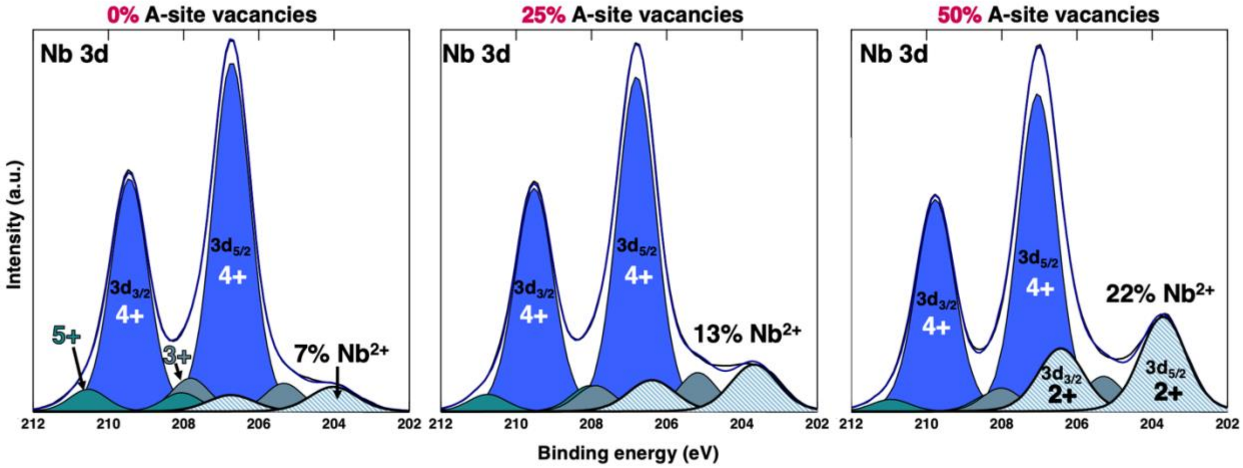

Figure S19. XPS fittings for the Nb 3d spectra of three films with increasing Sr vacancies, demonstrate a corresponding increase in Nb^{2+} concentration

Note 11: X-ray absorption near edge structure (XANES)

XANES measurements were conducted using an easyXAFS300+ spectrometer (Renton, WA), operating in fluorescence mode with a Mo X-ray tube set at 40 kV and 30 mA. A minimum of 75 scans were performed for the Sr K-edge (16,105 eV) and the W L_3 -edge (10,207 eV). Data analysis was carried out with Athena software from the Demeter package[26], while peak fitting was performed using Larch[27]. Ta metal (9,881 eV) was employed for energy calibration of the W L_3 -edge. The XANES results are illustrated in Figure S20. The Sr K-edge, in Figure S20(a), corresponds to Sr in a high coordination environment, closely resembling strontium carbonate or doped aragonite, where Sr exhibits 9-fold coordination[28]. Figure S20(b) shows the W L_3 absorption edge, which corresponds to an electronic transition from the $2p_{3/2}$ to an empty 5d state. The splitting of the d states is observed in the second derivative of the W L_3 spectra Figure S20(c). The energy difference between the two minima in this derivative, measured to be 3.7 eV, indicates a distorted octahedral environment for W, based on comparisons with similar systems[29]. Further analysis involved fitting the W L_3 white line with two Lorentzian functions centered at the energy values of the minima, as shown in Figure S20(b). The areas under these Lorentzian functions reflect the absorption intensities of the t_{2g} and e_g orbitals. Compared to other materials with distorted W octahedral environments, the absorption intensity of the t_{2g} orbital is lower than expected. Additionally, the E_0 shift of the W L_3 absorption edge was used to estimate the average oxidation state. The E_0 shift, calculated as 6.4 eV, suggests that the majority oxidation state of W in this sample is 6+.

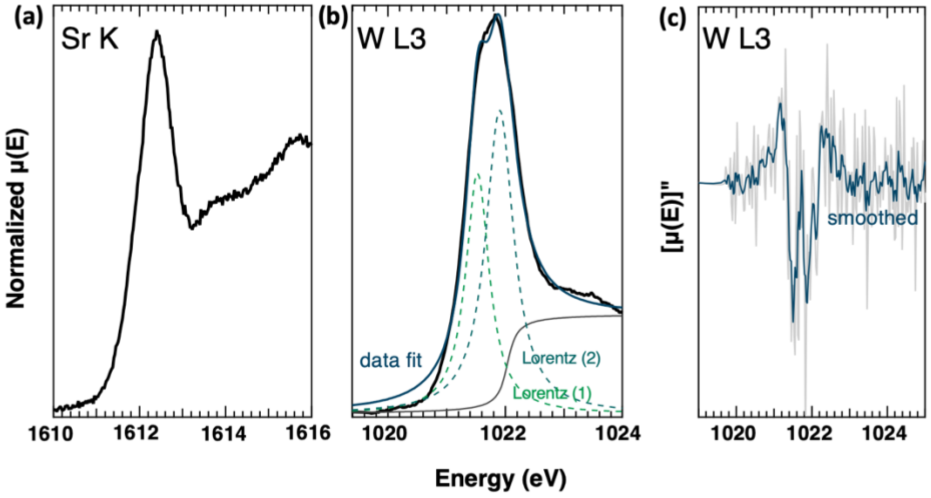

Figure S20. XANES Sr K absorption edge (a) and WL3 absorption edge (b). The second derivative of WL3 is plotted in (c).

Note 12: Optical coefficient for $Sr_{0.95}BO_3$ films on LSAT

The real part of the dielectric function (ε1) in Figure S21(a) indicates a screened plasma frequency for the high-entropy films well below the visible range, around 1.33 eV, which is comparable to $SrVO_3$ and red-shifted compared to Sr_xNbO_3. The imaginary part (ε2) and the extinction coefficient (k) in Figure S21(b) and (c) suggest very low absorption in the visible and UV regimes, with a sharp increase at higher photon energies attributed to $E_{O2p\text{-}t2g}$ interband transitions. Figure S21 (d) shows the extinction coefficient (k), calculated from ultraviolet-visible (UV-Vis) spectroscopy in transmission mode. The extinction coefficient (k) obtained from both ellipsometry in reflection mode and UV-Vis in transmission mode shows remarkable agreement, strengthening our confidence in the measurements and fittings. $Sr_{0.95}BO_3$ samples exhibit slightly lower k values compared to Sr_xNbO_3 particularly at lower energies, suggesting higher optical transmission in the high-entropy samples.

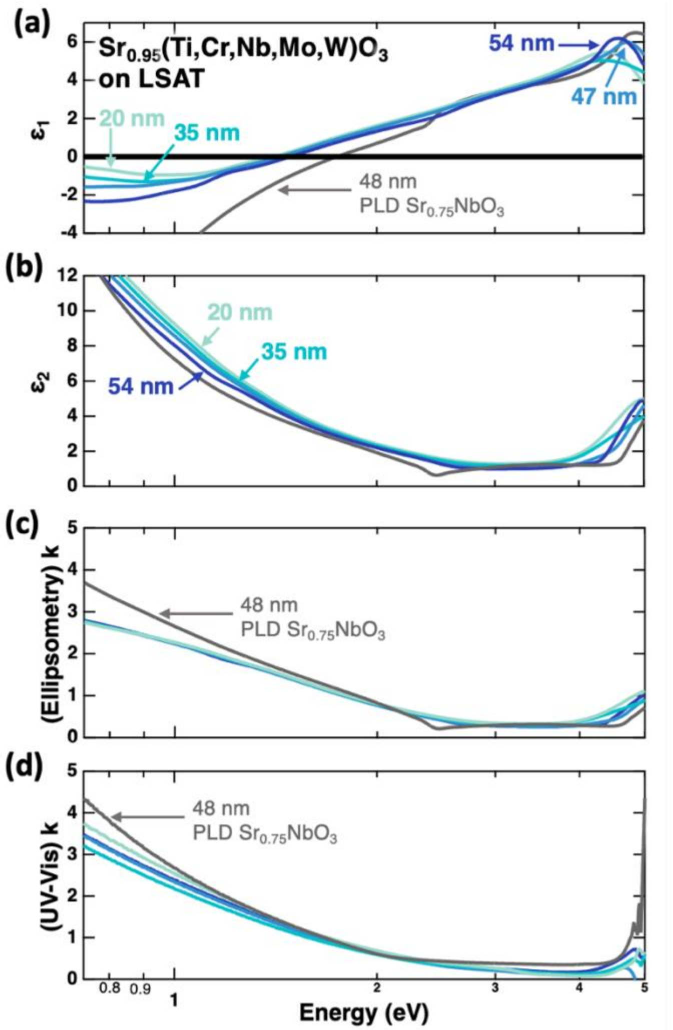

Figure S21. (a)-(c) summarizes the ellipsometry optical properties of $Sr_{0.95}BO_3$ in comparison to PLD grown Sr_xNbO_3 (this study) and (d) shows the extinction coefficient calculated from UV-Vis.

Note 13:

Everything matters: Sr_xBO_3 resistivity distribution over three years and 200 samples

Due to the high metastability of Sr_xBO_3, the thin film growth conditions are highly sensitive and have a significant impact on the resulting electronic properties of the samples. For example, given the oxygen-rich nature of the targets and the tendency of Mo and W to favor the ABO_4 structure, it is critical to maintain an optimal flow of reducing gas (in this case, Ar) to promote the formation of the ABO_3 phase. However, even a slight excess in gas flow could lead to the reduction of W and Mo. Similar considerations apply to parameters such as growth rate, time on the heater, growth temperature, and laser fluence.

Figure S22 shows a histogram illustrating the resistivity distribution of 200 samples grown under varied conditions. Films with room-temperature resistivity below 1000 μΩ·cm were produced under specific conditions: a substrate temperature of 850°C measured by thermocouple and 700°C measured by external pyrometer, an Ar flow of 40 sccm and pressure of 50 mTorr (the vacuum chamber background pressure is ~8 x 10^{-8} Torr), laser fluence of 1.4 J/cm², and a growth rate of 22 nm/min. Post-growth, the films were quenched to ambient conditions within six minutes after the final laser pulse to minimize any post-synthesis annealing effects.

Growth Parameter	Range Explored
Substrate temperature (thermocouple)	650 – 950 °C
Laser fluence	0.8 – 1.5 J/cm^2
Growth rate	7 – 30 nm/min
Ar gas flow	0 – 50 sccm
Ar gas pressure	1 – 100 mTorr
Total time in the chamber	2 hrs – 12 min

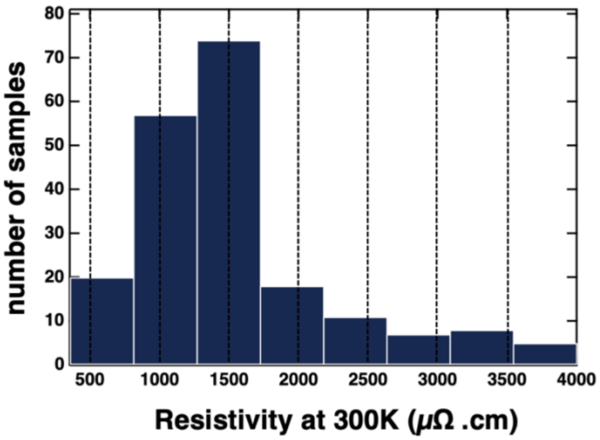

Figure S22. Sr_xBO_3 room temperature resistivity histogram over three years and 200 samples

Note 14:

Short-range chemical ordering in $SrBO_3$

Methods

Density Functional Theory (DFT) calculations for the cluster expansion were carried out using version 6.4.2 of the Vienna Ab initio Simulation Package VASP software[17], within the spin-polarized generalized gradient approximation (GGA) and the Perdew-Burke-Ernzerhof (PBE) parametrization.[30] Hubbard U corrections were applied to the 3d-orbitals of Cr (U=3.7 eV), Mo (U=4.38 eV), and W (U=6.2 eV), and energies were calculated following the methodology employed by the Materials Project (database release v2021.11.10).[31,32] Ionic cores were represented using Projector Augmented Wave (PAW) pseudopotentials[17,33,34], and the energy cutoff for the plane-wave basis set was 680 eV. The Brillouin zone was sampled using a Monkhorst-Pack grid with a k-spacing of 0.22 Å^{-1}. Atomic configurations were relaxed at zero pressure, using a maximum force threshold of 10 meV·Å^{-1}, while allowing ionic positions, cell shape, and cell volume to relax. To minimize the Pulay stress, relaxation runs were repeated until the volume change between two consecutive runs was less than 1%. Finally, the enthalpy of mixing per formula unit was computed as

$$\Delta H_{\mathrm{mix}}^{\mathrm{DFT}} = \frac{E_{conf}^{DFT} - \sum_M N_M E_{\mathrm{SrMO_3}}^{DFT}}{\sum_M N_M} \quad (1),$$

where E_{conf}^{DFT} and $E_{\mathrm{SrMO_3}}^{DFT}$ are energies of the perovskite compounds calculated using DFT, and N_M is the number of atoms of type M in the perovskite configuration denoted “conf”.

The cluster expansion (CE) method was employed to evaluate the enthalpies of mixing of configurations containing thousands of atoms.

This method uses an orthogonal basis of cluster functions to describe cluster interactions in a crystal structure where $\gamma_{\alpha_i}(\sigma_i)$ is an orthogonal basis function of a cluster, $\boldsymbol{\alpha}$, within a specific configuration, $\boldsymbol{\sigma}$, and the product is calculated over all the lattice sites. The mixing energies are then modeled as a linear combination of the cluster functions (eqn. 2), where $J_{\boldsymbol{\alpha}}$ are the cluster interactions:

$\Gamma_\alpha(\boldsymbol{\sigma}) = \prod_i \gamma_{\alpha_i}(\sigma_i)$ (2) and $\Delta H_{mix}^{CE}(\boldsymbol{\sigma}) = \sum_{\boldsymbol{\alpha}} J_{\boldsymbol{\alpha}} \Gamma_{\boldsymbol{\alpha}}(\boldsymbol{\sigma})$ (2).

The effective cluster interactions were extracted using Automatic Relevance Determination Regression (ARDR), from a database of 1478 atomic configurations relaxed within DFT. Starting from the orthorhombic unit cell of the Pnma space group, containing 4 formula units, symmetrically unique configurations over the composition space $\mathrm{Sr}Ti_{x_{Ti}}Cr_{x_{Cr}}Nb_{x_{Nb}}Mo_{x_{Mo}}W_{x_W}O_3$ ($\sum_M x_M = 1$) were generated using the structure enumeration module within the Integrated Cluster Expansion Toolkit Python package. We used orthorhombic unit cells to avoid inconsistencies between energies evaluated in small and large cells with cubic symmetry. All 175 configurations containing 4 formula units were included. Among structures made of 8 formula units, 1303 binary $\mathrm{Sr}X_{x_X}Y_{x_Y}O_3$ and quinary $\mathrm{Sr}Ti_{x_{Ti}}Cr_{x_{Cr}}Nb_{x_{Nb}}Mo_{x_{Mo}}W_{x_W}O_3$

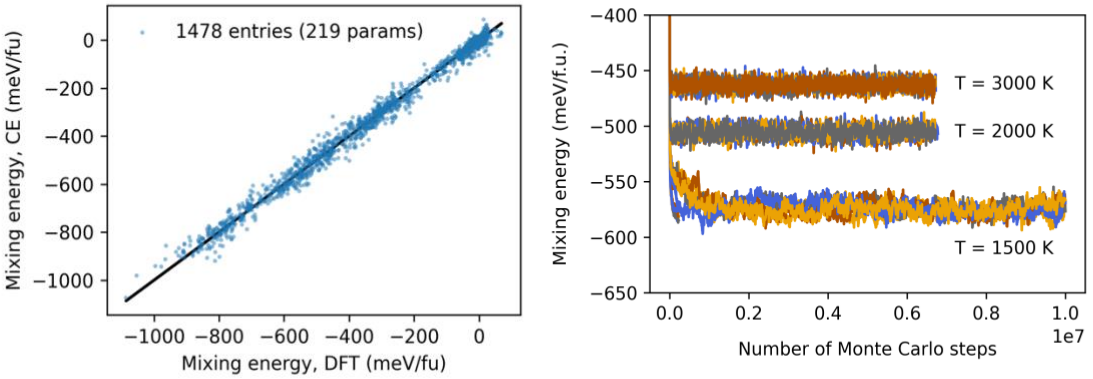

Figure S23. (a) Parity plot comparing the DFT and CE energies of the 1478 configurations used to fit the CE model (made of 219 non-zero parameters after ARDR). The out-of-sample Root Mean Square Error is equal to 6.5 meV/atom and the coefficient of determination, R^2, is 0.99, and (b) Evolution of mixing energy as a function of Monte Carlo step number for the equimolar $Sr(Ti,Cr,Nb,Mo,W)_{1/5}O_3$ configurations. 5 independent samples, with an initially random distribution of transition metal elements, were equilibrated at 1500 K, 2000 K, and 3000 K.

configurations were randomly sampled and relaxed. The CE model was built using all pair and triplet clusters such that the largest distance between two atoms in a cluster is less than 8.5 Å for pairs (140 pairs) and 6.5 Å for triplets (640 triplets). Cluster model predictions are reported in Figure S23(a). They are in reasonable agreement with the DFT calculations. Using the fitted cluster expansion model, 5 independent $Sr(Ti,Cr,Nb,Mo,W)_{1/5}O_3$ atomic configurations, each containing 5000 atoms, were equilibrated at 1500 K, 2000K, and 3000K using Metropolis Monte Carlo simulations within the canonical ensemble – the evolution of mixing energy at different temperatures with Monte Carlo steps is depicted in Figure S23(b).[38]

Comment on equilibration temperature

We employ the Warren-Cowley parameter[39], $w_{\alpha\beta,n}$, to quantify chemical ordering, defined as

$$w_{\alpha\beta,n} = 1 - \frac{p_{\alpha\beta,n}}{c_\beta} \quad (3)$$

where $p_{\alpha\beta,n}$ is the probability of finding a type β atom with c_β concentration in the nth nearest neighbor shell of a type α atom. In the absence of chemical ordering, $p_{\alpha\beta,n} = c_\beta$ yielding $w_{\alpha\beta,n} = 0$. Therefore, nonzero $w_{\alpha\beta,n}$ suggests chemical ordering, with negative values indicating clustering tendencies and positive values suggesting repulsive interactions between species α and β.

Although the films are not equilibrated at a controlled temperature during deposition, they grow as crystalline solids. This suggests that they undergo some degree of energy relaxation via thermally activated processes, such as vacancy diffusion, and supports assessing tendencies for chemical ordering in the out-of-equilibrium films using thermally equilibrated atomistic configurations. Additionally, temperature modulates the amplitude of ordering, but preserves the overall trends, as demonstrated by the Warren-Cowley analyses at different temperatures reported in Figure S24. Therefore, the discussion of ordering does not depend on the choice of temperature.

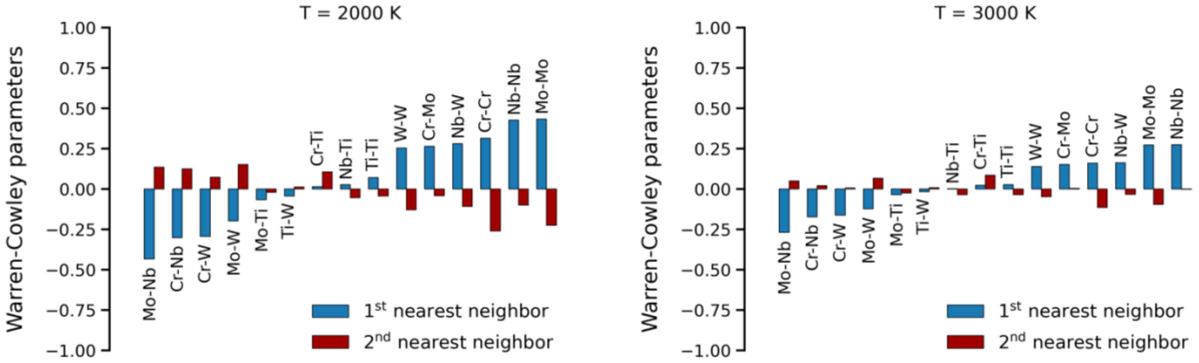

Figure S24. Warren-Cowley parameters for pairs of species in the first and second nearest neighbor shells. As temperature increases, the amplitudes of Warren-Cowley parameters decrease. On the contrary, their ordering, from most attractive to most repulsive pairs, is almost unaltered.

Note 15:

Chemical disorder and flexibility coupled with high crystalline quality: Unlocking broader research opportunities beyond our focus composition

Although the compositions presented in this manuscript already represent highly metastable systems, we successfully extended these limits even further, stabilizing three additional groups of compositions into single-phase cubic perovskite structures (Figure S25). The first group features four-component B-site systems, including a non-equimolar variant with an increased W content of 40%. The second group introduces La^{3+} doping (25%) on the A-site in a five-component B-site system, significantly increasing resistivity. The third group explores replacing Ti with Mn, challenging solubility and metastability, since Mn typically favors the hexagonal perovskite polymorph at lower temperatures. The motivation behind the first two groups—high-W-content and La^{3+}-doped compositions—originates from preliminary results suggesting large spin-Hall angles compared to $SrIrO_3$[13], making these compositions promising for spintronic applications. Conversely, Mn substitution not only tests metastability limits but also explores functionalities predicted to arise from Mn incorporation, such as ferrimagnetic or multiferroic responses.

With 20,349 potential equimolar five-cation $SrBO_3$ combinations derived from transition metals: Ti, V, Cr, Mn, Fe, Co, Ni, Cu, Zn, Zr, Nb, Mo, Ru, Rh, Pd, Hf, Ta, W, Re, Os, and Ir, extending disorder to the A-site[40] and varying molar ratios yields virtually infinite possibilities. Additionally, exploring related Ruddlesden-Popper structures ($A_{n+1}B_nO_{3n+1}$, n=∞) further expands this vast compositional space. Therefore, targeted, intelligent composition design is crucial for identifying technologically impactful compositions—such as Fe-modulated ferrimagnetism[41] or Cu-driven high-temperature superconductivity[42]—highlighting an expansive frontier awaiting discovery and technological integration.

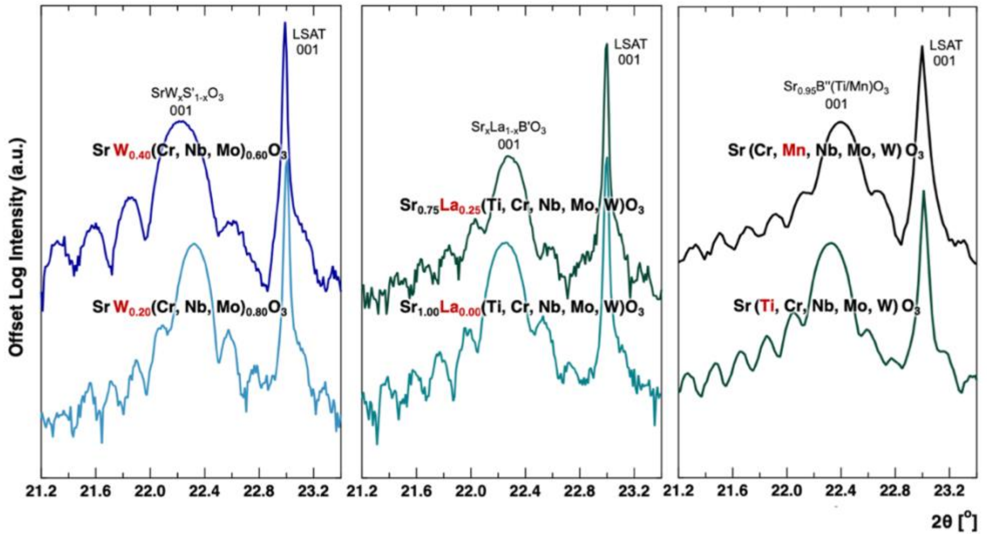

Figure S25. Additional three sets of metastable compositions were stabilized in a single-phase cubic perovskite structure. The first set, shown on the left, consists of four-component systems on the B-site, with the top film being a non-equimolar composition containing 40% W. The middle panel illustrates a five-component system with 25% La^{3+} on the A-site. On the right, an example is presented where Ti is replaced with Mn as a fifth B-site cation.

References

1. Ophus, C., Ciston, J. & Nelson, C. T. Correcting nonlinear drift distortion of scanning probe and scanning transmission electron microscopies from image pairs with orthogonal scan directions. *Ultramicroscopy* **162**, 1–9 (2016).
2. Kotsonis, G. N. *et al.* High-entropy oxides: Harnessing crystalline disorder for emergent functionality. *J. Am. Ceram. Soc.* **106**, 5587–5611 (2023).
3. Rost, C. M. Entropy-stabilized oxides. *Nat. Commun.* **6**, 8485 (2015).
4. Almishal, S. S. I. *et al.* Untangling individual cation roles in rock salt high-entropy oxides. *Acta Mater.* **279**, 120289 (2024).
5. Almishal, S. S. I. *et al.* Order evolution from a high-entropy matrix: Understanding and predicting paths to low-temperature equilibrium. *J. Am. Ceram. Soc.* e20223 doi:10.1111/jace.20223.
6. Almishal, S. S. I. *et al.* Untangling individual cation roles in rock salt high-entropy oxides. Preprint at https://doi.org/10.48550/arXiv.2405.07918 (2024).
7. Mazza, A. R. *et al.* Designing magnetism in high entropy oxides. *Adv. Sci.* **9**, 2200391 (2022).
8. Yoo, S. *et al.* Efficient data processing using tunable entropy-stabilized oxide memristors. *Nat. Electron.* **7**, 466–474 (2024).
9. Sarkar, A. *et al.* High entropy oxides for reversible energy storage. *Nat. Commun.* **9**, 3400 (2018).
10. Bérardan, D., Franger, S., Meena, A. K. & Dragoe, N. Room temperature lithium superionic conductivity in high entropy oxides. *J. Mater. Chem. A* **4**, 9536–9541 (2016).
11. Spurling, R. J. *et al.* Dielectric properties of disordered A6B2O17 (A = Zr; B = Nb, Ta) phases. *J. Am. Ceram. Soc.* **n/a**,.
12. Manipatruni, S. *et al.* Scalable energy-efficient magnetoelectric spin–orbit logic. *Nature* **565**, 35–42 (2019).
13. Yoo, M.-W. *et al.* Large intrinsic anomalous Hall effect in SrIrO3 induced by magnetic proximity effect. *Nat. Commun.* **12**, 3283 (2021).
14. Kang, K., Li, T., Sohn, E., Shan, J. & Mak, K. F. Nonlinear anomalous Hall effect in few-layer WTe2. *Nat. Mater.* **18**, 324–328 (2019).
15. Varignon, J., Bibes, M. & Zunger, A. Origin of band gaps in 3d perovskite oxides. *Nat. Commun.* **10**, 1658 (2019).
16. Wang, Z., Malyi, O. I., Zhao, X. & Zunger, A. Mass enhancement in 3d and s-p perovskites from symmetry breaking. *Phys. Rev. B* **103**, 165110 (2021).
17. Kresse, G. & Furthmüller, J. Efficient iterative schemes for ab initio total-energy calculations using a plane-wave basis set. *Phys. Rev. B* **54**, 11169–11186 (1996).
18. Furness, J. W., Kaplan, A. D., Ning, J., Perdew, J. P. & Sun, J. Accurate and Numerically Efficient r2SCAN Meta-Generalized Gradient Approximation. *J. Phys. Chem. Lett.* **11**, 8208–8215 (2020).

19. Ong, S. P. *et al.* Python Materials Genomics (pymatgen): A robust, open-source python library for materials analysis. *Comput. Mater. Sci.* **68**, 314–319 (2013).

20. Larsen, A. H. *et al.* The atomic simulation environment—a Python library for working with atoms. *J. Phys. Condens. Matter* **29**, 273002 (2017).

21. Ganose, A. M., Jackson, A. J. & Scanlon, D. O. sumo: Command-line tools for plotting and analysis of periodic* ab initio* calculations. *J. Open Source Softw.* **3**, 717 (2018).

22. Zhu, B., Kavanagh, S. R. & Scanlon, D. easyunfold: A Python package for unfolding electronic band structures. *J. Open Source Softw.* **9**, 5974 (2024).

23. Henkelman, G., Arnaldsson, A. & Jónsson, H. A fast and robust algorithm for Bader decomposition of charge density. *Comput. Mater. Sci.* **36**, 354–360 (2006).

24. Roth, J. *et al.* Sputtered SrxNbO3 as a UV-Transparent Conducting Film. *ACS Appl. Mater. Interfaces* **12**, 30520–30529 (2020).

25. Denev, S. A., Lummen, T. T. A., Barnes, E., Kumar, A. & Gopalan, V. Probing Ferroelectrics Using Optical Second Harmonic Generation. *J. Am. Ceram. Soc.* **94**, 2699–2727 (2011).

26. Ravel, B. & Newville, M. ATHENA, ARTEMIS, HEPHAESTUS: data analysis for X-ray absorption spectroscopy using IFEFFIT. *J. Synchrotron Radiat.* **12**, 537–541 (2005).

27. Newville, M. Larch: An Analysis Package for XAFS and Related Spectroscopies. *J. Phys. Conf. Ser.* **430**, 012007 (2013).

28. Finch, A. & Allison, N. Coordination of Sr and Mg in calcite and aragonite. *Mineral. Mag. - Min. MAG* **71**, 539–552 (2007).

29. Yamazoe, S., Hitomi, Y., Shishido, T. & Tanaka, T. XAFS Study of Tungsten L1- and L3-Edges: Structural Analysis of WO3 Species Loaded on TiO2 as a Catalyst for Photooxidation of NH3. *J. Phys. Chem. C - J PHYS CHEM C* **112**, 6869–6879 (2008).

30. Perdew, J. P., Burke, K. & Ernzerhof, M. Generalized Gradient Approximation Made Simple. *Phys. Rev. Lett.* **77**, 3865–3868 (1996).

31. Jain, A. *et al.* Formation enthalpies by mixing GGA and GGA+U calculations. *Phys. Rev. B* **84**, 045115 (2011).

32. Jain, A. *et al.* A high-throughput infrastructure for density functional theory calculations. *Comput. Mater. Sci.* **50**, 2295–2310 (2011).

33. Kresse, G. & Joubert, D. From ultrasoft pseudopotentials to the projector augmented-wave method. *Phys. Rev. B* **59**, 1758–1775 (1999).

34. Blöchl, P. E. Projector augmented-wave method. *Phys. Rev. B* **50**, 17953–17979 (1994).

35. Hart, G. L. W. & Forcade, R. W. Generating derivative structures from multilattices: Algorithm and application to hcp alloys. *Phys. Rev. B* **80**, 014120 (2009).

36. Hart, G. L. W. & Forcade, R. W. Algorithm for generating derivative structures. *Phys. Rev. B* **77**, 224115 (2008).

37. Ångqvist, M. *et al.* ICET – A Python Library for Constructing and Sampling Alloy Cluster Expansions. *Adv. Theory Simul.* **2**, 1900015 (2019).

38. Metropolis, N., Rosenbluth, A. W., Rosenbluth, M. N., Teller, A. H. & Teller, E. Equation of State Calculations by Fast Computing Machines. *J. Chem. Phys.* **21**, 1087–1092 (1953).

39. Cowley, J. M. Short-Range Order and Long-Range Order Parameters. *Phys. Rev.* **138**, A1384–A1389 (1965).

40. Yan, J. *et al.* Orbital degree of freedom in high entropy oxides. *Phys. Rev. Mater.* **8**, 024404 (2024).

41. Regmi, B. *et al.* Apparent ferrimagnetism in Sr(Fe0.2Mn0.2Co0.2Ti0.2V0.2)O3 high-entropy oxide perovskite thin films. *AIP Adv.* **14**, 025023 (2024).

42. Mazza, A. R. *et al.* Searching for superconductivity in high entropy oxide Ruddlesden–Popper cuprate films. *J. Vac. Sci. Technol. A* **40**, 013404 (2021).
